# Supplementary material for: Two Interlinked Bistable Switches Govern Mitotic Control in Mammalian Cells
Source: Curr Biol. 2018 Dec 3;28(23):3824–3832.e6. doi: 10.1016/j.cub.2018.09.059 (PMC6287978; doi:10.1016/j.cub.2018.09.059)
Supplement: Document S1. Figures S1–S4 [file mmc1.pdf]

Current Biology, Volume *28*

Supplemental Information

Two Interlinked Bistable Switches Govern Mitotic Control in Mammalian Cells

Scott Rata, Maria F. Suarez Peredo Rodriguez, Stephy Joseph, Nisha Peter, Fabio Echegaray Iturra, Fengwei Yang, Anotida Madzvamuse, Jan G. Ruppert, Kumiko Samejima, Melpomeni Platani, Monica Alvarez-Fernandez, Marcos Malumbres, William C. Earnshaw, Bela Novak, and Helfrid Hochegger

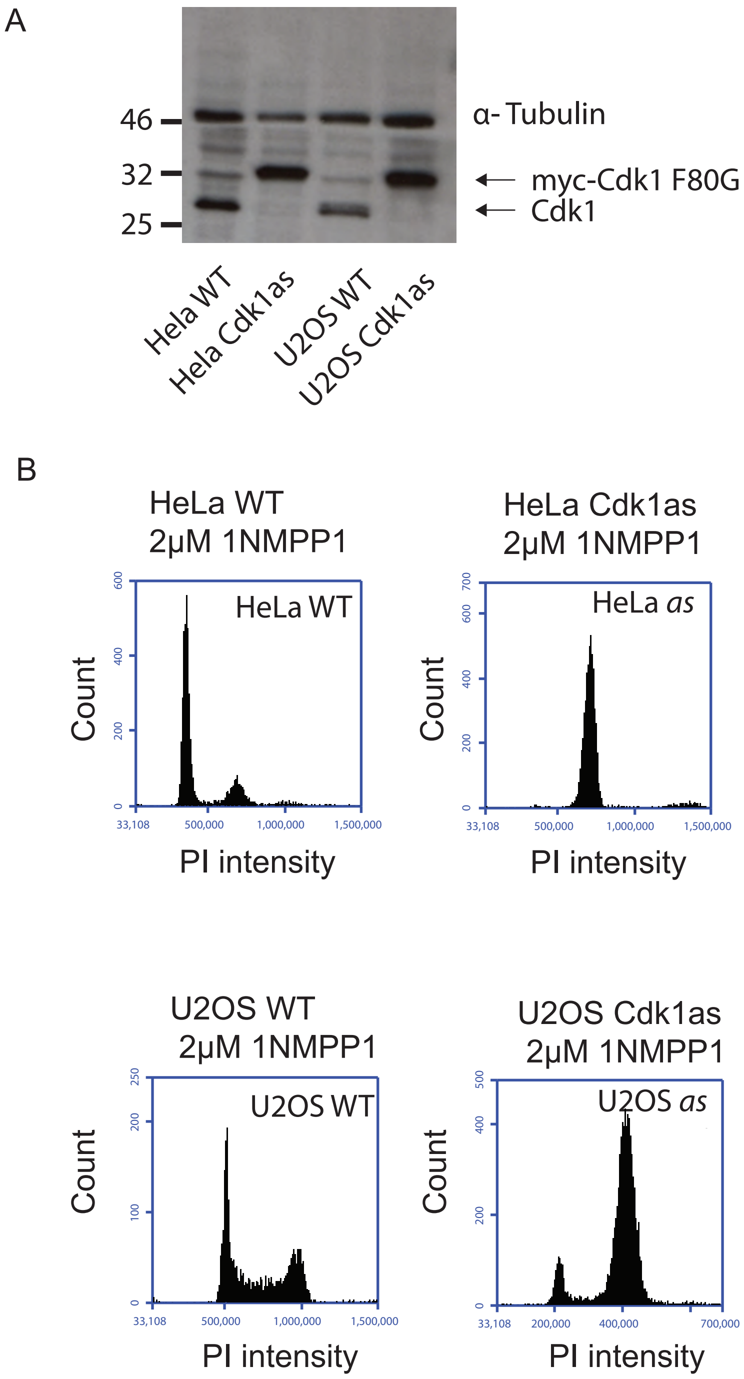

Figure S1 Characterisation of cdk1as cells (Related to Figure 2)
(A) Immuno-blots confirming generation of cdk1as HeLa and U2OS cells. (B) PI staining and FACS analysis of HeLA and U2OS WT and cdk1as cells after 20 hour treatment with 2 µM 1NMPP1.

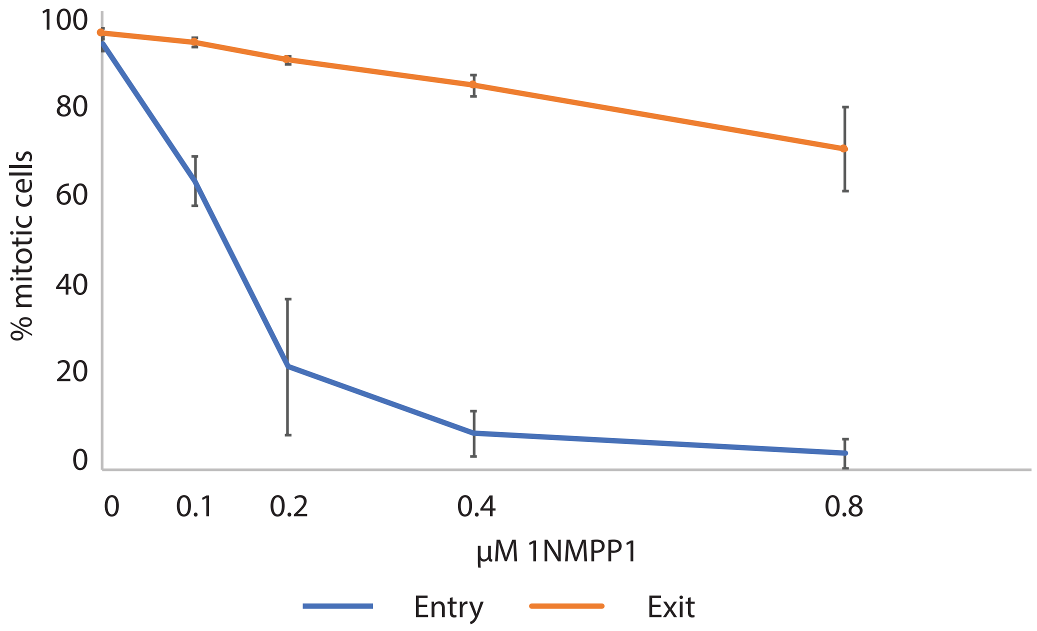

Figure S2 Bistability assay in U2OS cdk1as cells (Related to Figure 2)
Experiments performed as described in Figure 2D in U2OS cdk1as cells (means and stdv of 3 biological repeats are shown, with N=100 per repeat)

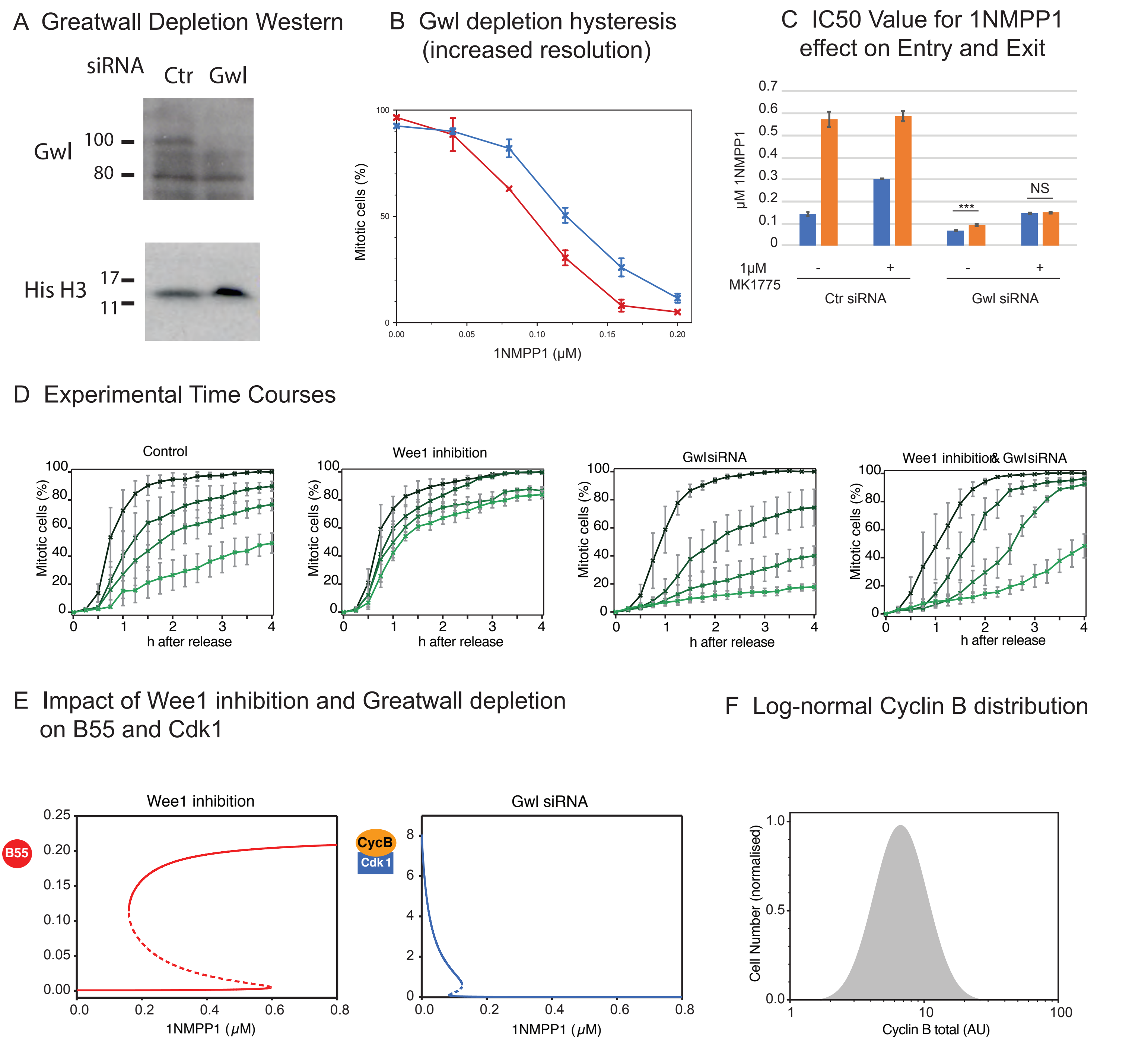

Figure S3 Data supporting bistability assays (related to Figure 3)

(A) Immuno-blots confirming Gwl siRNA knockdown efficiency. HeLa cdk1as cells were probed 48 hours after siRNA transfection. (B) Mitotic entry and exit curves of cdk1as HeLa cells depleted of Gwl at increased resolution of 1NMPP1 concentration. Mean of two repeats plotted, with error bars indicating standard deviation.
(C) Midpoint values of 1NMPP1 response based on experiments in Figure 3B and linear interpolation. Means of 3–4 biological repeats are plotted, with error bars indicating standard deviation.
Statistical significance was assessed by the one-tailed student's t-test.
(D) Dynamics of mitotic entry at increasing 1NMPP1 concentrations (0, 0.1, 0.2, and 0.4 µM) from live-cell imaging analysis in cells subjected to indicated treatment. Graphs represent the mean of three independent experiments and error bars indicate standard error. (E) Simulated signal-response curves for PP2A:B55 activity (left) and Cdk1 activity (right) as a function of Cdk1 inhibitor for the cases of Wee1 inhibition and Gwl depletion, respectively. The bistability of these enzymes gives rise to the hysteresis of Cdk1/PP2A:B55 substrate phosphorylation in Figure 3C (F) Estimated Cyclin B distribution that was used to model the population analysis data shown in Figure 3D, with median 8.18 AU and standard deviation 4.31 AU.

Figure S4

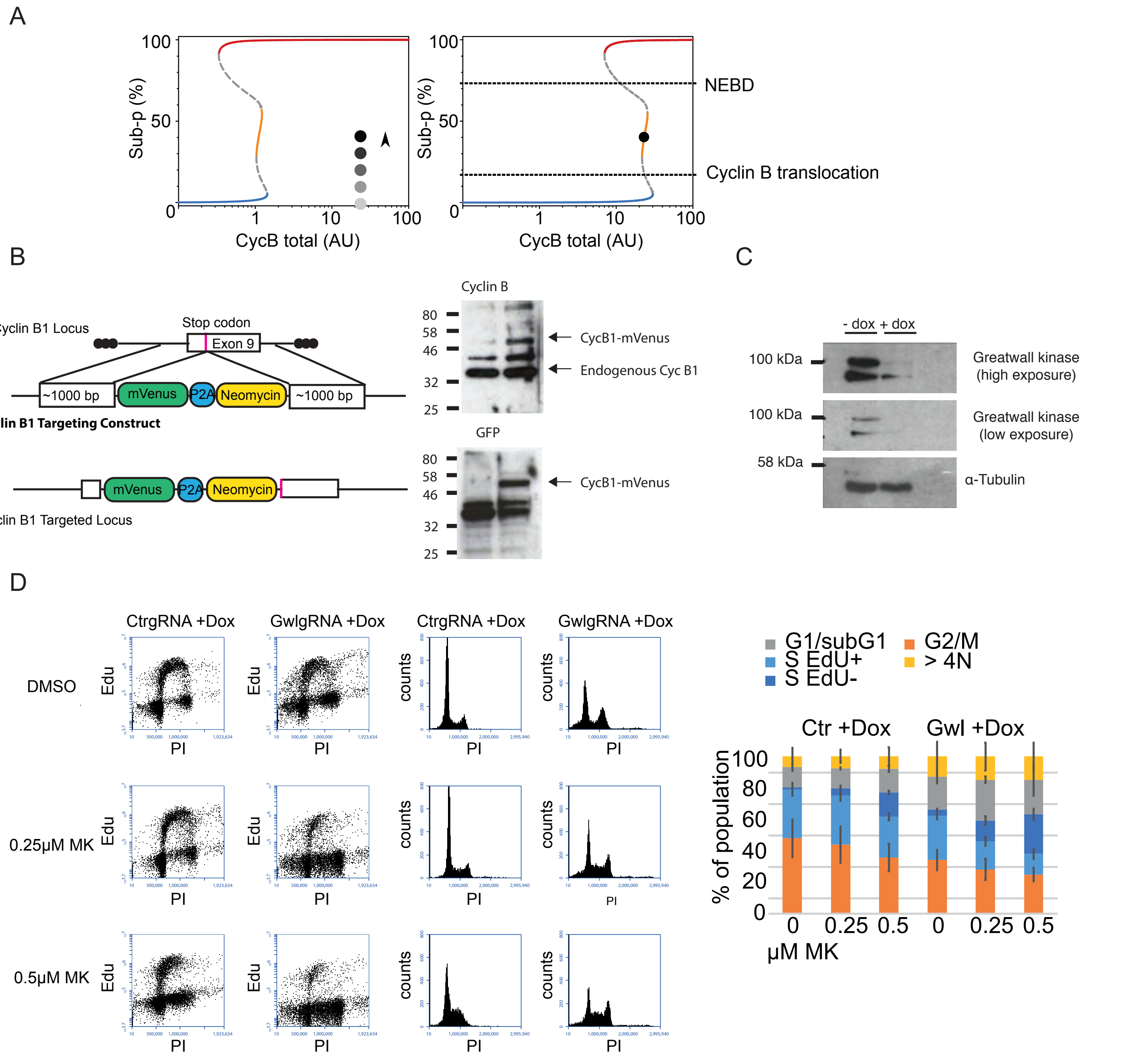

Figure S4 Data supporting prophase experiments and Wee1/Greatwall synergy (Related to Figure 4)
(A) Signal-response curve for cells (filled circles) released from G2 block (left) and after Cdk1 inhibitor (0.5 μM) addition (right). Addition of the right amount of inhibitor at the right time captures some cells at the intermediate steady state. (B) Strategy and confirmation of generation of CyclinB-GFP tagged HeLa cdk1as cells by immune-blotting
(C) Depletion of Greatwall kinase in MDA MB 231 cells three days after induction of GwlgRNA/Cas9. (D) Representative examples and quantification of FACS profiles after PI/Edu staining of Ctr gRNA/Cas9 or Greatwall gRNA/Cas9 expressing MDA MB 231 cells (4 days after addition of Doxycyclin). Cells were treated with indicated concentration of MK1775 for the last 24 hours before Edu labelling. Shown are the means of three biological repeats (N=10000 per repeat), Error bars represent the STDV of three independent experiments.
