# Supplementary material for: Two Interlinked Bistable Switches Govern Mitotic Control in Mammalian Cells
Source: Curr Biol. 2018 Dec 3;28(23):3824–3832.e6. doi: 10.1016/j.cub.2018.09.059 (PMC6287978; doi:10.1016/j.cub.2018.09.059)
Supplement: Document S2. Article plus Supplemental Information [file mmc6.pdf]

Current Biology

Report

Two Interlinked Bistable Switches Govern Mitotic Control in Mammalian Cells

Graphical Abstract

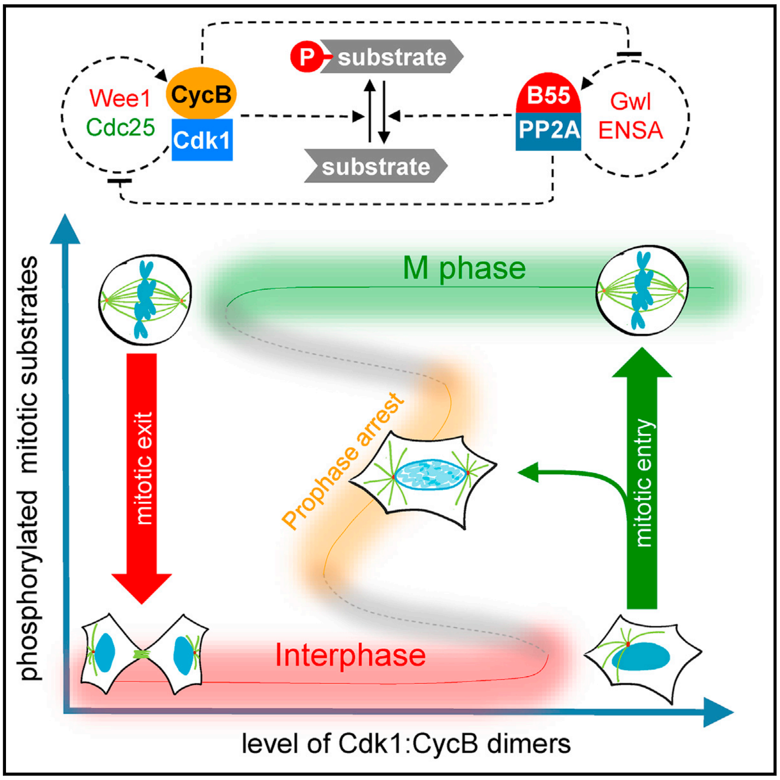

Authors

Scott Rata,
Maria F. Suarez Peredo Rodriguez,
Stephy Joseph, ...,
William C. Earnshaw, Bela Novak,
Helfrid Hochegger

Correspondence

bela.novak@bioch.ox.ac.uk (B.N.),
h.hochegger@sussex.ac.uk (H.H.)

In Brief

Rata et al. combine quantitative assays with mathematical modeling to show that hysteresis of the mitotic switch emerges from mutual effects of Cdk1 and its counteracting phosphatase, PP2A:B55, on each other's auto-activation loops. Their work also reveals an intermediate but hidden steady state between interphase and M phase.

Highlights

- The transitions between interphase and M phase in human cells show robust hysteresis
- Auto-regulation of both Cdk1 and PP2A:B55 contributes to the hysteresis response
- Constitutively active Cdk1 and PP2A:B55 make mitotic transitions blurred
- The switch-like transition from interphase to M phase can be arrested in prophase

Rata et al., 2018, Current Biology *28*, 3824–3832
December 3, 2018 © 2018 The Authors. Published by Elsevier Ltd.
https://doi.org/10.1016/j.cub.2018.09.059

Current Biology
Report

Two Interlinked Bistable Switches Govern Mitotic Control in Mammalian Cells

Scott Rata,[1,7,8] Maria F. Suarez Peredo Rodriguez,[2,7] Stephy Joseph,[2] Nisha Peter,[2] Fabio Echegaray Iturra,[2] Fengwei Yang,[3] Anotida Madzvamuse,[4] Jan G. Ruppert,[5] Kumiko Samejima,[5] Melpomeni Platani,[5] Monica Alvarez-Fernandez,[6] Marcos Malumbres,[6] William C. Earnshaw,[5] Bela Novak,[1,*] and Helfrid Hochegger[2,9,*]
[1]Department of Biochemistry, University of Oxford, South Park Road, Oxford OX1 3QU, UK
[2]Genome Damage and Stability Centre, University of Sussex, Science Park Road, Brighton BN1 9RQ, UK
[3]Department of Chemical and Process Engineering, University of Surrey, 388 Stag Hill, Guildford GU2 7JP, UK
[4]Department of Mathematics, University of Sussex, Science Park Road, Brighton BN1 9QH, UK
[5]Wellcome Trust Centre for Cell Biology, University of Edinburgh, Max Born Crescent, Edinburgh EH9 3BF, UK
[6]Spanish National Cancer Research Centre, Melchor Fernandez Almagro, Madrid E28029, Spain
[7]These authors contributed equally
[8]Present address: Department of Systems Biology, Harvard Medical School, Boston, MA 02115, USA
[9]Lead Contact
*Correspondence: bela.novak@bioch.ox.ac.uk (B.N.), h.hochegger@sussex.ac.uk (H.H.)
https://doi.org/10.1016/j.cub.2018.09.059

SUMMARY

Distinct protein phosphorylation levels in interphase and M phase require tight regulation of Cdk1 activity [1, 2]. A bistable switch, based on positive feedback in the Cdk1 activation loop, has been proposed to generate different thresholds for transitions between these cell-cycle states [3–5]. Recently, the activity of the major Cdk1-counteracting phosphatase, PP2A:B55, has also been found to be bistable due to Greatwall kinase-dependent regulation [6]. However, the interplay of the regulation of Cdk1 and PP2A:B55 *in vivo* remains unexplored. Here, we combine quantitative cell biology assays with mathematical modeling to explore the interplay of mitotic kinase activation and phosphatase inactivation in human cells. By measuring mitotic entry and exit thresholds using ATP-analog-sensitive Cdk1 mutants, we find evidence that the mitotic switch displays hysteresis and bistability, responding differentially to Cdk1 inhibition in the mitotic and interphase states. Cdk1 activation by Wee1/Cdc25 feedback loops and PP2A:B55 inactivation by Greatwall independently contributes to this hysteretic switch system. However, elimination of both Cdk1 and PP2A:B55 inactivation fully abrogates bistability, suggesting that hysteresis is an emergent property of mutual inhibition between the Cdk1 and PP2A:B55 feedback loops. Our model of the two interlinked feedback systems predicts an intermediate but hidden steady state between interphase and M phase. This could be verified experimentally by Cdk1 inhibition during mitotic entry, supporting the predictive value of our model. Furthermore, we demonstrate that dual inhibition of Wee1 and Gwl kinases causes loss of cell-cycle memory and synthetic lethality, which could be further exploited therapeutically.

RESULTS AND DISCUSSION

When cells enter M phase, a burst of morphological changes occurs, resulting in a profound reorganization of various cellular compartments in preparation for chromosome segregation and cell division. These changes are driven by Ser/Thr phosphorylation of over a thousand proteins, predominantly by cyclin-dependent kinase 1 (Cdk1) in complex with cyclin B (CycB) [7, 8]. Thus, Cdk1:CycB activation is the crucial event leading to mitosis, and the dynamics of this process have long been a focus of theoretical exploration. A key activation step of this kinase is the removal of Wee1/Myt1-dependent inhibitory phosphorylations of Cdk1 at Thr14/Tyr15 by Cdc25 phosphatases. Both inhibitory kinases and activating phosphatases are linked via positive feedback with Cdk1, creating bistability in Cdk1 activity with respect to total CycB (Figure 1A) [3]. The bistable switch, well-known in engineering [9, 10], creates two distinct states, corresponding to interphase and M phase, without allowing the cell to come to rest in intermediate transitional states. There are distinct thresholds for mitotic entry and mitotic exit, so that once a cell accumulates enough CycB and commits to mitotic entry, it will only exit mitosis at a lower CycB level. This difference in thresholds provides robustness of the M phase state and prevents the cell from flipping back to the interphase state in the noisy cellular environment. Bistability of the mitotic switch system was confirmed in *Xenopus* extracts [4, 5] but has not been directly tested in intact mammalian cells. Moreover, the original Novak/Tyson mitotic switch model presumed a constitutive Cdk1-counteracting phosphatase, whose identity was unknown at the time. In recent years, however, it has become apparent that Cdk1-counteracting protein phosphatases (PP1 and PP2A) are also under stringent regulation [11, 12]. The best example for this is PP2A with its B55 regulatory subunit (PP2A:B55), which is tightly regulated by Greatwall (Gwl) kinase [13] via its substrates ENSA and ARPP19 that become potent

 © 2018 The Authors. Published by Elsevier Ltd.
This is an open access article under the CC BY license (http://creativecommons.org/licenses/by/4.0/).

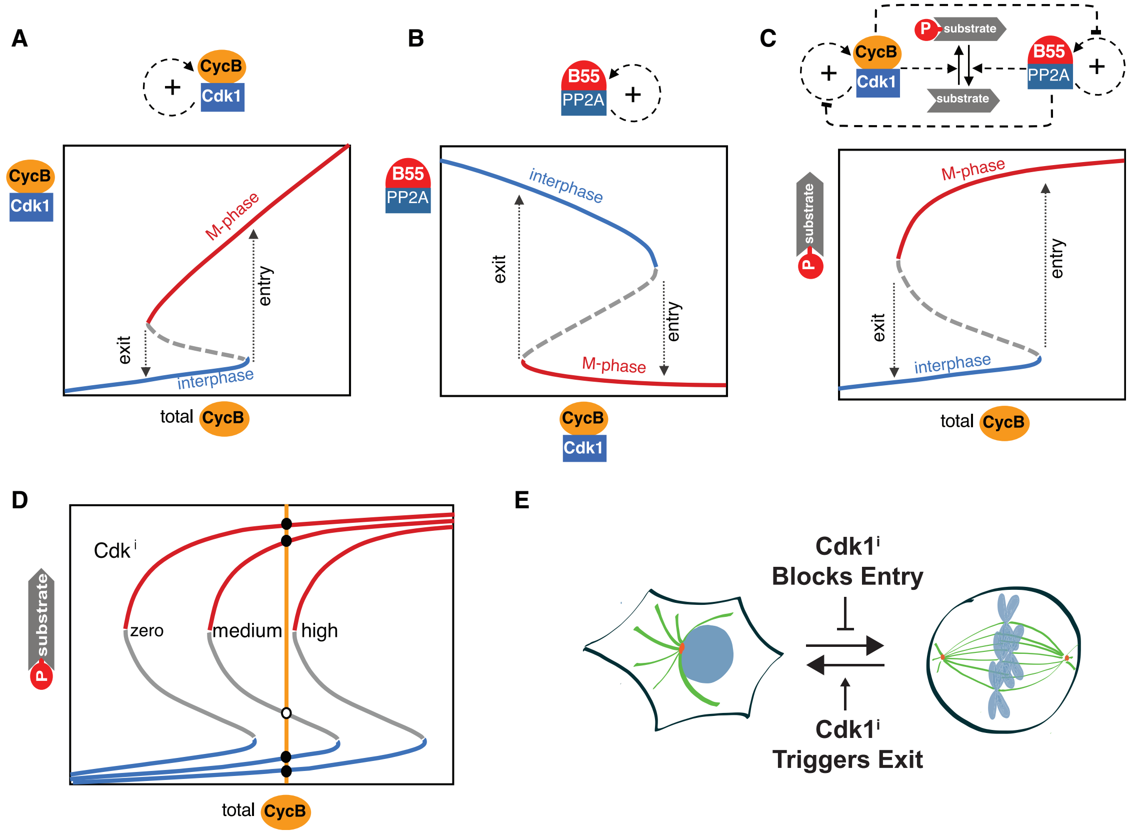

Figure 1. Bistable Switches of Mitotic Control

(A–C) Schematic signal-response (SR) diagram for Cdk1 auto-activation (A), PP2A:B55 feedback regulation (B), and mitotic substrate phosphorylation by interlinked kinase–phosphatase switches (C).

(D) Model of the hysteresis assay based on Cdk1 inhibition. The Cdk1 inhibitor shifts the SR curve to the right in a concentration-dependent manner: more inhibitor is required to induce mitotic exit (right curve) than to block mitotic entry (middle curve).

(E) Summary: Cdk1 inhibitor blocks mitotic entry and promotes mitotic exit.

PP2A:B55 inhibitors upon phosphorylation [14, 15]. Gwl itself is activated by Cdk1-dependent phosphorylation [16], which is reversed by PP1 [17–19] and PP2A:B55 [6, 20], and the latter creates a mutual antagonism. Reconstitution of the Gwl-ENSA-PP2A:B55 pathway *in vitro* confirmed these interactions and revealed that PP2A:B55 has a bistable activity with respect to Cdk1 activity [6] (Figure 1B). What remains to be determined is how these two bistable switches of Cdk1:CycB and PP2A:B55 are interlinked during interphase–M phase transitions in the context of the somatic mammalian cell cycle. Given that Cdk1 influences PP2A:B55 activity via Gwl and PP2A:B55 negatively regulates Cdk1 via Wee1 and Cdc25 [21], one can imagine that the two feedback systems might reinforce each other, thereby increasing the robust separation of interphase and M phase states (Figure 1C). However, Gwl depletion and genetic deletion in mammalian cells results only in minor delays in the G2/M transition and does not interfere with establishing the mitotic state and initiating cell division [22–24]. Thus, the precise contributions of the Cdk1 activation and PP2A:B55 inhibition feedback network to the G2/M switch system remain to be determined.

We set out to establish a quantitative assay for interphase–M phase bistability in human cells. A key feature of mitotic bistability is hysteresis—mitotic entry requires a larger cyclin B level than that required to block the reverse transition at mitotic exit (Figure 1C). We reasoned that hysteresis of mitotic transitions could also be quantitatively assessed by exposing cells to increased concentrations of a Cdk1 inhibitor that shifts the S-shaped substrate phosphorylation curve to the right (Figure 1D). If the mitotic switch is bistable and shows hysteresis, the threshold concentration of Cdk1 inhibitor required to block mitotic entry (θ_{entry}) should be smaller than the one needed to induce mitotic exit (θ_{exit}) at a given cyclin B level (Figure 1D).

To test this prediction, we used an analog-sensitive mutation in Cdk1 (cdk1as) that allows specific and reversible inhibition with the ATP analog 1NM-PP1 [25, 26] (Figures 1E, S1A, and S1B). Thus, measuring the 1NM-PP1 concentrations required to prevent entry into mitosis and to trigger mitotic exit should allow us to determine θ_{entry} and θ_{exit}. To simplify this assay, we performed G2/M synchronization of cdk1as cells (i.e., arresting them in G2 by 1NM-PP1 treatment and releasing them from G2 into mitosis) and used proteasome inhibition throughout the experiment to ensure constant cyclin B levels (see Figure 2A for experimental setup and Figure 2B and Video S1 for an example of a mitotic entry and exit experiment).

These highly synchronous mitotic entry and exit experiments allow a quantitative assessment of the Cdk1 inhibitor thresholds

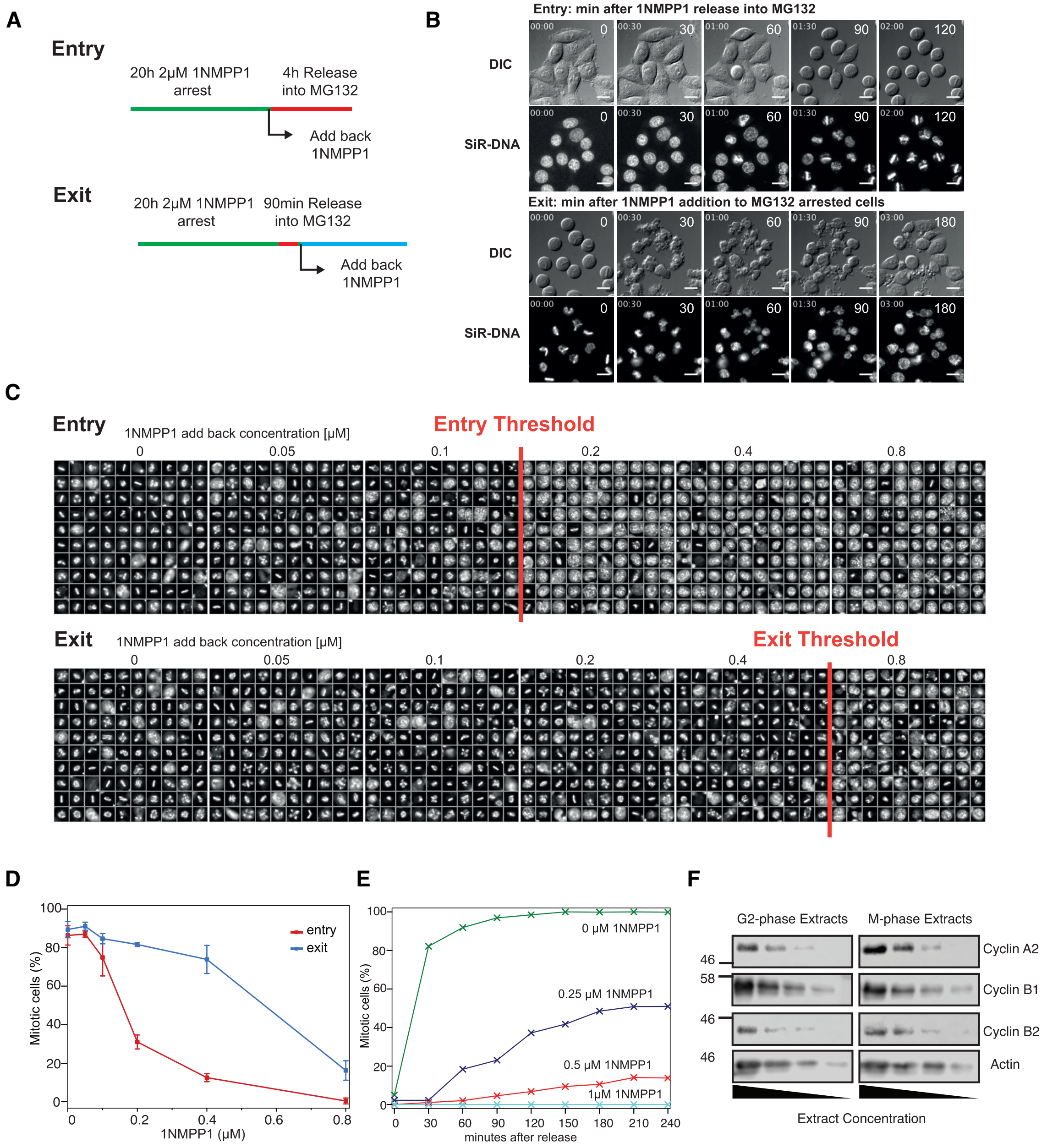

Figure 2. Hysteresis of Mammalian Mitotic Control

(A) Experimental protocol to measure threshold 1NM-PP1 concentrations at constant CycB levels in cdk1as cells (see Figure S1 for characterization of cdk1as cells and STAR Methods for details of how they were generated).

(B) Still images from time-lapse videos (see also Video S1) of HeLa cdk1as cells released from 20-hr 1NM-PP1 arrest (entry, top two panels) or treated with 2 μM 1NM-PP1 90 min after mitotic arrest in MG132 (exit, bottom two panels). Time in minutes from release into MG132 (top panels) or from 1NM-PP1 add-back (bottom panels) is shown. Scale bars indicate 10 μm.

(C) Galleries of nuclei from HeLa cdk1as cells, imaged 4 hr after entry and exit treatments (1NM-PP1 concentrations in μM as indicated above the panels). For each panel, one hundred nuclei were randomly chosen by Olympus SCANR software. Interphase nuclei appear rounded and enlarged; mitotic nuclei are rod-shaped and condensed. The red lines indicate the borders upon which 1NM-PP1 is becoming active for either entry or exit.

(D) Quantifications of entry and exit experiments in HeLa cdk1as cells (see Figure S2 for similar results with U2OS cdk1as cells). The values are means of three biological repeats (N = 100 per repeat), and error bars indicate SD.

(E) Temporal dynamics of mitotic entry at increasing 1NM-PP1 concentrations from live-cell imaging analysis.

(F) Levels of mitotic cyclins in G2- and M-arrested cells analyzed by immuno-blotting with indicated antibodies. Each panel shows four steps of a serial 1:1 dilution of extracts.

on these cell-cycle transitions. To obtain these measurements, we released HeLa cdk1as cells into increasing concentrations of 1NM-PP1 and tested for mitotic entry. For exit experiments, cells were released into 1NMPP-free medium and subsequently forced out of the mitotic state by increasing 1NM-PP1 concentrations. To assess the percentage of cells in mitosis and interphase following a dose-response curve with 1NM-PP1, we used an endpoint assay 4 hr after 1NM-PP1 addition and scored for interphase or mitosis by the morphology of Hoechst-stained DNA as shown in Figure 2C. Representative galleries of Hoechst-stained single cells show a clear threshold to block entry at 0.2 μM 1NM-PP1. In contrast, a similar induction of mitotic exit was only achieved at doses between 0.4 and 0.8 μM 1NM-PP1, suggesting a 2- to 4-fold change in entry and exit thresholds. Quantification of these data revealed a half-maximal inhibitory concentration (IC_{50}) change from 143 nM to 573 nM between entry and exit experiments (Figure 2D). A similar result was also obtained in U2OS cdk1as cells (Figure S2A). We also followed cells entering mitosis by live-cell imaging and quantified the percentage of mitotic cells over time using an automated detection algorithm (see STAR Methods). The rate of entry into mitosis declined with increasing inhibitor concentrations (Figure 2E), with the mitotic population reaching saturation 2 or 3 hr following 1NM-PP1 addition for each condition. We noted that cells appeared less sensitive to 1NM-PP1 when imaged on the fluorescence microscope, suggesting that 1NM-PP1 might be affected by light exposure. This made a direct comparison between endpoint and live-cell assays difficult, but we proceeded with both strategies to confirm thresholds and saturation. The interpretation of these experiments requires identical mitotic cyclin levels during mitotic entry and exit experiments, and this was verified by immuno-blotting (Figure 2F).

Based on the hysteresis assay shown in Figure 2, we aimed to establish a mathematical model to simulate this experiment. This model was designed to test the contribution of phosphatase and kinase regulation to bistability and to perform parameter fitting to satisfy all experimental conditions. To this end, we used nonlinear ordinary differential equations describing the feedback regulation of Cdk1 and PP2A:B55 (see Figure 3A for the basic wiring diagram; for details on the equations and parameters, see STAR Methods). To obtain experimental parameters for this model and to determine the contribution of individual feedbacks to bistability, we measured the hysteresis effect in HeLa cdk1as cells following Wee1 inhibition and Gwl depletion (Figure S3A) both individually and in combination (Figure 3B). Wee1 inhibition only had a mild effect on hysteresis, shifting the mitotic entry curve toward increased 1NM-PP1 doses (midpoint around 300 nM), and did not significantly affect the mitotic exit curve. Gwl depletion had a more pronounced effect, shifting both entry and exit curves toward lower 1NM-PP1 (midpoints around 67 nM and 92 nM, respectively; Figures S3B and S3C). The effect of Gwl depletion on the entry threshold was partially reverted by Wee1 inhibition, and we observed a complete collapse of hysteresis in these conditions (IC_{50}s for entry and exit at 147 nM and 150 nM, respectively; Figures 3B and S3C). Time course experiments of mitotic entry under the various conditions confirmed that saturation was reached between three to 4 hr (Figure S3D). These data suggest that both PP2A:B55 and Cdk1 autoregulation contribute to bistability, which is only lost if both feedback systems become compromised. If the regulation of Cdk1 did not contribute to the bistability of the system, we would not expect to observe hysteresis with Gwl depletion, and if the regulation of PP2A:B55 did not contribute to the overall bistability, we would not expect to observe hysteresis with Wee1 inhibition.

The kinetic constants of the model were determined by fitting the model to the experimental data, with initial estimates from the literature, where available [27]. To determine whether a cell is in interphase or M phase, we included a generic Cdk1/PP2A:B55 substrate in the model and set a threshold phosphorylation level for the interphase-M phase boundary. Based on immunofluorescence assays (see Figure 4E), we attributed this threshold to be 30% maximal substrate phosphorylation. Plotting the predicted steady-state phosphorylation level of the generic Cdk1/PP2A:B55 substrate against 1NM-PP1 concentration (Figure 3C) suggests that the model captures the salient features of the data. There is a clear difference between the 1NM-PP1 doses needed to block mitotic entry and forcing mitotic exit in the control case. When addition of Wee1 inhibitor is simulated, the 1NM-PP1 threshold of mitotic entry is increased, and that of exit is unchanged. This latter feature of the model requires that PP2A:B55 inhibits amplification of Cdk1 activity, which together with Cdk1-dependent Greatwall activation makes the kinase–phosphatase feedback systems mutually inhibitory, as proposed on Figure 1C. The hysteresis in this case is due to bistable PP2A:B55 activity (Figure S3E). With Gwl depletion, the model has reduced bistability and both thresholds are reduced. PP2A:B55 is modeled to be constitutively active with this perturbation and dephosphorylates the regulators of Cdk1 and the generic Cdk1/PP2A:B55 substrate. The hysteresis in this case is due to bistable Cdk1 activity (Figure S3E). A combination of Wee1 inhibition and Gwl depletion eliminates bistability in the model. We also simulated variance among the population of cells, assuming a log-normal distribution of total cyclin B (Figure S3F), with good agreement to the experimental data (Figure 3D).

A surprising prediction of our mathematical model for the G2/M switch is the existence of a third stable steady state in between interphase and mitosis. This steady state did not appear in previous models but emerged as a feature of our model after fitting to all experimental conditions. The state is not observable during normal mitotic progression; therefore, using the model, we devised an experiment to test its existence. Our model suggests that cells can be captured in this hypothetical intermediate stable state if Cdk1 activity is partially reduced during the G2-M transition (Figure S4A). To test this prediction, we used endogenously tagged CyclinB1-mVenus (Figure S4B) as a prophase marker (i.e., nuclear localization of cyclin B and intact nuclear envelope [NE]) and followed cells that were released from 1NM-PP1 and re-exposed to increasing 1NM-PP1 concentrations 25 min after release (Figure 4A). Prophase cells with nuclear cyclin B that lost sufficient amounts of Cdk1 activity are expected to re-export cyclin B to the cytoplasm, as previously reported [28] (Figure 4B, left panels), and insufficient Cdk1 inhibition would allow the cells to continue to M phase marked by nuclear envelope breakdown (NEBD) (Figure 4B, middle panels). We expected from the model that a fraction of cells should remain in prophase in this experiment, and we could indeed

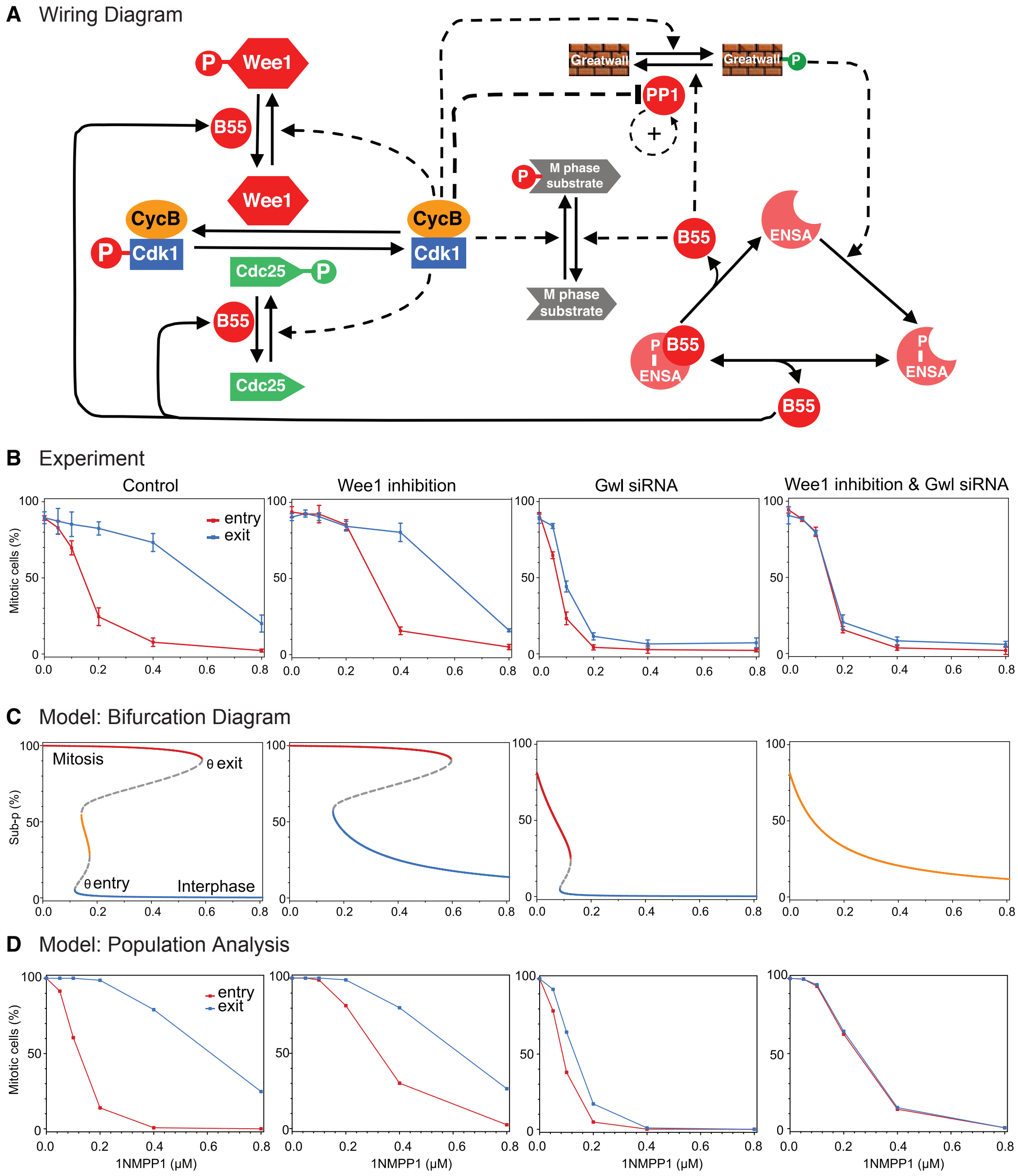

Figure 3. Contribution of Cdk1 and PP2A:B55 Feedbacks to Hysteresis

(A) Wiring diagram of the mitotic switch that forms the basis of the modeling for this paper.

(B) Percentage of cells entering into (red) and staying in (blue) mitosis at different Cdk1 inhibitor concentrations. Control (left column), Wee1-inhibited (second column), Gwl-depleted (third column; see Figure S3A for confirmation of depletion and Figure S3B for a higher resolution of 1NM-PP1 concentrations for this condition), and both Wee1-inhibited and Gwl-depleted (right column) cells are shown, with means of 3 or 4 biological repeats (N = 100 per repeat) and errors bars indicating SD (see Figure S3C for comparison of IC_{50}s for the different experimental conditions and Figure S3D for time course data).

(C) Signal-response curves for mitotic substrate phosphorylation in single cells as a function of inhibitor concentration. Steady-state substrate phosphorylation is high (red), low (blue), or intermediate (orange).

(D) Simulated population response of mitotic cells assuming log-normal CycB distribution among individual cells (see Figure S3F for our estimation of cyclin B concentration in the population, on which this model was based).

observe cells that kept nuclear cyclin B and remained rounded up but did not undergo NEBD (Figure 4B, right panels; see also Video S2).

We quantified the nuclear-cytoplasmic ratio of cyclin B in cells with intact nuclei. This resulted in a clear separation in a “back to G2 (i.e. loss of nuclear cyclin B)” versus “prophase (i.e. cyclin B

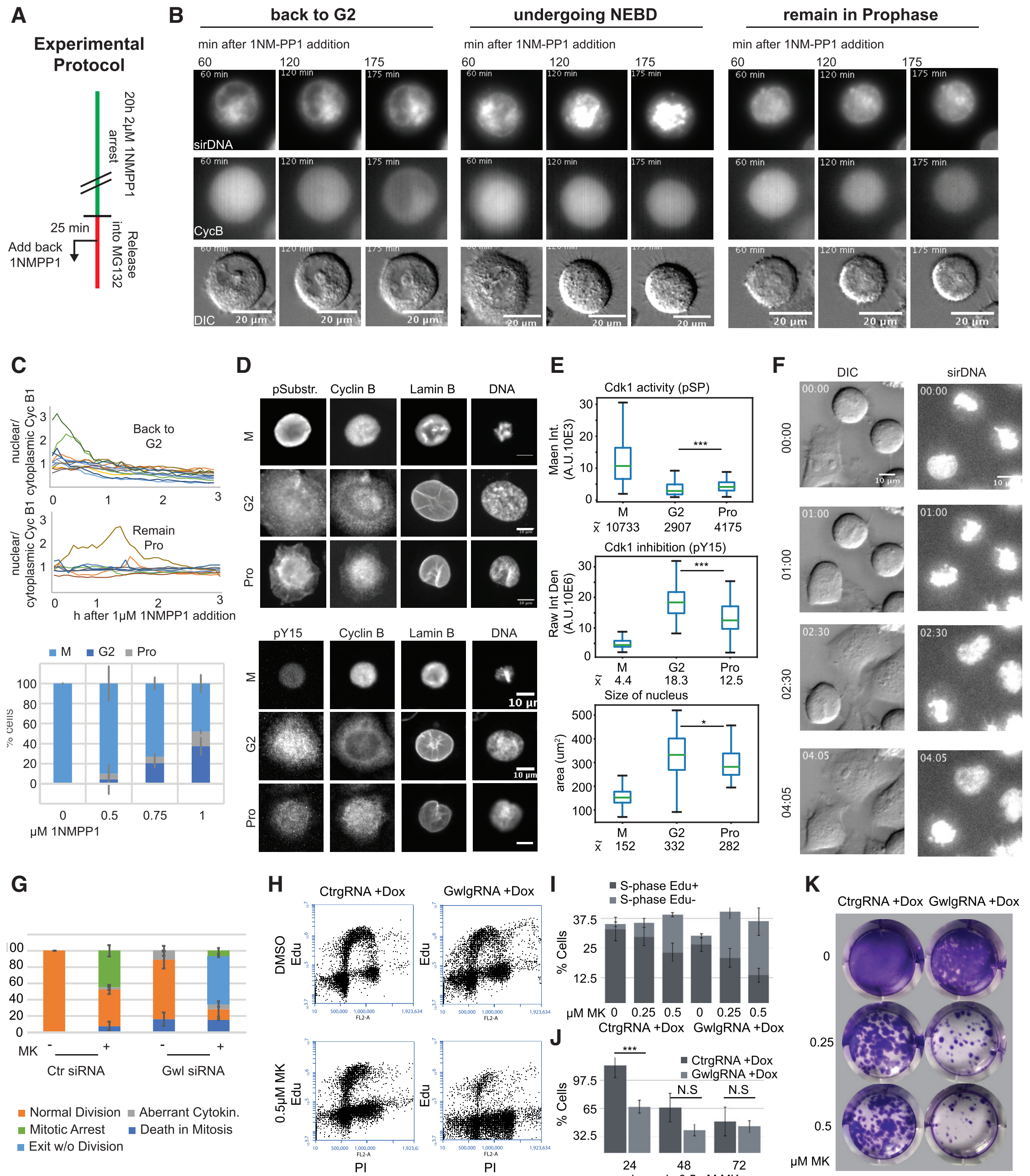

Figure 4. Testing Predictions of the Model

(A) Explanation of the synchronization protocol to obtain cells arrested in prophase (see Figure S4A for a simulation of this experiment based on the model).

(B) Images of cyclin B1-GFP-tagged HeLa cdk1as cells released from 1NM-PP1 arrest and retreated with 1 μM 1NM-PP1 25 min after release (see Figure S4B for characterization of the GFP knockin cells). Time after re-addition of 1NM-PP1 is indicated in minutes; scale bars correspond to 20 μm. Left panels show cells moving back to G2 (chromosome decondenses and cyclin B is exported and cells flatten); middle panels show cells moving to mitotic arrest (chromosome condensation, nuclear envelope breakdown, and cell rounding); right panels show cells that remain in prophase (chromosomes partially condensed, cyclin B remains nuclear, and cells remain rounded up). See also Video S2.

(C) Measurements of cytoplasmic versus nuclear cyclin B1 ratio in single cells (top panels). Quantification of cell-cycle states in cells treated as described in (B) 4 hr after 1NM-PP1 addition (bottom). N > 20 in three independent experiments, error bars indicate SD.

(D) Representative images of cells fixed 4 hr after 1NM-PP1 re-addition and stained by immuno-fluorescence with indicated antibodies. Scale bar, 10 μM.

(E) Quantification of Cdk1 substrate phosphorylation, Cdk1 Y15 phosphorylation, and nuclear surface of cells in the G2/M and prophase steady states (data are from three independent experiments; N > 40 per experimental repeat; error bars indicate SD; p values were assessed using an independent two-sample t test; significant [***p < 0.001; *p < 0.05] p values are indicated).

(F) Images of HeLa cells progressing through mitosis after combined Gwl depletion and Wee1 inhibition. Time intervals are indicated in hr:min; scale bars indicate 10 μm; see also Videos S3 and S4.

(legend continued on next page)

remains nuclear)" population (Figure 4C, top two panels). The prophase population was only observed when Cdk1 inhibitor was added back following release from the G2 arrest and increased with increasing inhibitor dose to about 10% at 1 μM 1NM-PP1 (Figure 4C, bottom panel). We further characterized this prophase steady state by immuno-fluorescence (Figure 4D), probing for cyclin B, lamin, Cdk1 substrate phosphorylation, and Cdk1 Y15 phosphorylation. In cell populations that were released from 1NM-PP1 and re-treated with 1 μM 1NM-PP1, we could readily identify a population of prophase cells 4 hr after release from the G2 arrest, and we did not observe similar prophase cells 4 hr after release without re-addition of 1NM-PP1. These cells were characterized by nuclear cyclin B, partially condensed DNA, yet intact nuclear envelope as judged by lamin B staining (Figure 4D). Compared with G2 and M phase cells, the prophase cells showed intermediate levels of Cdk1 activity and Y15 dephosphorylation, as predicted by the model, and displayed a decreased nuclear area, suggesting an intermediate state of chromosome compaction (Figure 4D; quantification in Figure 4E).

We next aimed to investigate whether loss of bistability has consequences for mitotic progression and cell proliferation that could ultimately be of therapeutic benefit. We first analyzed the effects of loss of combined Gwl and Wee1 inhibition on mitotic progression in asynchronously dividing HeLa cdk1as cells. A large fraction of cells lacking both Gwl and Wee1 activity readily entered mitosis but subsequently failed to stabilize the metaphase state, reverting to an interphase state without any visible attempt at chromosome segregation and cytokinesis (Figure 4F; quantification in Figure 4G; see Video S3). This response was unique to the combined loss of the Gwl/Wee1 condition and not observed in the controls that all exited mitosis with chromosome segregation and cleavage furrow formation (Figure 4H; Video S4). This exit was also in marked difference to spindle assembly checkpoint slippage, in which Gwl-depleted cells left the mitotic state after attempting chromosome segregation and cytokinesis (Video S4). This result suggests that loss of bistability does, indeed, result in a failure to stabilize the metaphase state and also in a failure to initiate progression through mitosis toward cell division and G1. To investigate whether the synergistic effects of Gwl/Wee1 double inactivation could be used to inhibit cell proliferation in a therapeutic setting, we analyzed cell-cycle progression and proliferation in the triple-negative breast cancer cell line MDA MB 231, where Gwl is depleted via doxycycline-inducible Cas9/gRNA [29] (Figure S4C). Edu labeling and fluorescence-activated cell sorting (FACS) analysis showed a significant reduction in the replicating S phase population 24 hr after treatment with 0.25 and 0.5 μM of the Wee1 inhibitor MK1775 when Gwl was co-depleted (Figures 4H, 4I, and S4D). A 24-hr pulse treatment with the Wee1 inhibitor also significantly reduced cell proliferation as judged by cell counts 6 days after treatment (Figure 4J) and colony formation assays (Figure 4K). This synergy dissipated in longer term treatments with Wee1 inhibitors where the toxicity of Wee1 inhibition alone appeared to become dominant (Figure 4J).

Our results demonstrate that the mitotic switch in somatic human cells is bistable, clarify how Cdk1 and PP2A regulation contribute to the establishment of this switch, and provide a comprehensive quantitative model for mitotic transitions. Our observations suggesting that two interlinked bistable switches work together to stably separate interphase and M phase could reflect a paradigm also acting in other cellular switch systems [30]. The network architecture of this switch allows transitions to be made between states with maximum theoretical efficiency [31]. Our model is of predictive value as demonstrated by the discovery and verification of a new steady state in prophase. Although this steady state appears to be an integral feature of the dynamical switch system, it has not to our knowledge been described previously, and its physiological relevance remains to be addressed. Indeed, RPE-1 cells have been reported to stall in late G2 phase with nuclear cyclin B following DNA damage for several hours before Cyclin degradation and exit into senescence [32, 33]. It is tempting to speculate that this arrest point may be related to the prophase steady state observed in our study. Our results also suggest that abrogating bistability leads to an unstable mitotic state and to loss of the irreversible progression toward cell division. Accordingly, double inhibition of both Gwl and Wee1 causes an additive effect on proliferation in breast cancer cells (Figures 4H–4K). This synthetic lethality due to loss of bistability could be exploited therapeutically once specific Gwl inhibitors become available.

STAR★METHODS

Detailed methods are provided in the online version of this paper and include the following:

- KEY RESOURCES TABLE
- CONTACT FOR REAGENT AND RESOURCE SHARING
- EXPERIMENTAL MODEL AND SUBJECT DETAILS
  - General cell line culture conditions
  - HeLa cdk1as cells (Human cervical cancer cell line, female)
  - HeLa cdk1as cells with GFP tagged endogenous Cyclin B

(G) Quantification of mitotic phenotypes from live-cell imaging in HeLa cells.

(H) Cell-cycle profiles of MDA MB 231 cells following inducible Gwl knockout (see Figure S4C for confirmation of depletion) and Wee1 inhibition based on Edu (replicating population) and PI (DNA content) staining and FACS analysis.

(I) Average values for Edu-positive and negative S phase populations of MDA MB 231 cells after Gwl depletion and or Wee1 inhibition. Values are from three independent experiments; N = 10,000 per experiment; error bars indicate SD (see Figure S4D for quantification of all cell-cycle phases).

(J) Proliferation assays following increasing time of Wee1 inhibition in MDA MB 231 cells following Ctr and Gwl gRNA/Cas9 expression. Cells were counted based on Hoechst staining and nuclear segmentation 6 days after MK1775 pulse treatment. Values are from three independent experiments; N > 500 per experiment; error bars indicate SD. p values were assessed using an independent two-sample t test. Significant (***$p < 0.001$) or non-significant ($p > 0.05$) p values are indicated.

(K) Colony-formation assays in MDA MB 231 cells following Ctr and Gwl gRNA/Cas9 expression and 24-hr incubation with MK1775 at the indicated concentrations. 5,000 cells were plated into each 6-well plate and stained and imaged 2 weeks after MK1775 pulse treatment.

See also Figure S4.

- ○ U2OS cdk1as cells (Human bone osteosarcoma epithelial cell, female)
- ○ MDA MB 231 Cells expressing inducible Greatwall-Cas9-gRNA (Human triple negative breast cancer cell line, female)

- METHOD DETAILS
  - ○ Modeling of mitotic entry and exit
  - ○ Mathematical modeling of the variation within a population of cells
  - ○ Estimation of the kinetic parameters of the model
  - ○ Preparation of total cell extracts, immunoblotting
  - ○ siRNA transfections
  - ○ FACS analysis
  - ○ Immunofluorescence and live cell imaging
  - ○ Bistability Assay
  - ○ Automated measurement of mitotic index in live cell experiments
- QUANTIFICATION AND STATISTICAL ANALYSIS

SUPPLEMENTAL INFORMATION

Supplemental Information includes four figures and four videos and can be found with this article online at https://doi.org/10.1016/j.cub.2018.09.059.

ACKNOWLEDGMENTS

We thank the members of the Novak and Hochegger labs and the BBSRC Bicycle consortium for discussion. Thanks to Drs. Francis Barr and Ricardo Nunes Bastos for sharing unpublished data with us. H.H. received funding from a senior CRUK research fellowship (C28206/A14499). S.R. was supported by the EPSRC with a studentship (EP/G03706X/1). M.F.S.P.R. was supported by a Mexico CONACyT scholarship, CVU 409236. A.M. was partially supported by the European Union's Horizon 2020 Research and Innovation Program under the Marie Sklodowska-Curie grant agreement no. 642866. A.M. is a Royal Society Wolfson Research Merit Award Holder supported by the Wolfson Foundation. F.Y. and A.M. acknowledge support from the Leverhulme Trust Research Project Grant (RPG-2014-149). Work in the Earnshaw Lab is funded by The Wellcome Trust (107022), of which W.C.E. is a Principal Research Fellow. J.G.R. was supported by the EU FP7 PloidyNet ITN (grant 607722). This work was supported by a BBSRC LoLa grant (BB/M00354X/1) to B.N. and H.H.

AUTHOR CONTRIBUTIONS

S.R. was responsible for conceptual work, modelling, parameter fitting, and all theoretical aspects of the paper and wrote the manuscript. M.F.S.P.R. performed the experiments in Figures 2, 3, and 4. S.J. set up the initial bistability assays and performed experiments in Figure 2. N.P. helped with experiments in Figure 2. F.E.I. helped with data analysis and image processing. A.M. and F.Y. built the algorithm to track mitotic cells by phase-contract imaging. J.G.R., K.S., M.P., and W.C.E. contributed the HeLa cdk1as cell line and helped with writing the manuscript. M.A.-F. and M.M. contributed unpublished MDA MB 231 cells with inducible Greatwall gRNA/Cas9. H.H. and B.N. devised the concepts of the modelling and experiments, supervised the first authors, and wrote the manuscript.

DECLARATION OF INTERESTS

The authors declare no competing interests.

Received: August 7, 2018
Revised: September 14, 2018
Accepted: September 26, 2018
Published: November 15, 2018

REFERENCES

1. Coudreuse, D., and Nurse, P. (2010). Driving the cell cycle with a minimal CDK control network. Nature *468*, 1074–1079.
2. Stern, B., and Nurse, P. (1996). A quantitative model for the cdc2 control of S phase and mitosis in fission yeast. Trends Genet. *12*, 345–350.
3. Novak, B., and Tyson, J.J. (1993). Numerical analysis of a comprehensive model of M-phase control in Xenopus oocyte extracts and intact embryos. J. Cell Sci. *106*, 1153–1168.
4. Pomerening, J.R., Sontag, E.D., and Ferrell, J.E., Jr. (2003). Building a cell cycle oscillator: hysteresis and bistability in the activation of Cdc2. Nat. Cell Biol. *5*, 346–351.
5. Sha, W., Moore, J., Chen, K., Lassaletta, A.D., Yi, C.-S., Tyson, J.J., and Sible, J.C. (2003). Hysteresis drives cell-cycle transitions in Xenopus laevis egg extracts. Proc. Natl. Acad. Sci. USA *100*, 975–980.
6. Mochida, S., Rata, S., Hino, H., Nagai, T., and Novák, B. (2016). Two bistable switches govern M phase entry. Curr. Biol. *26*, 3361–3367.
7. Dephoure, N., Zhou, C., Villén, J., Beausoleil, S.A., Bakalarski, C.E., Elledge, S.J., and Gygi, S.P. (2008). A quantitative atlas of mitotic phosphorylation. Proc. Natl. Acad. Sci. USA *105*, 10762–10767.
8. Daub, H., Olsen, J.V., Bairlein, M., Gnad, F., Oppermann, F.S., Körner, R., Greff, Z., Kéri, G., Stemmann, O., and Mann, M. (2008). Kinase-selective enrichment enables quantitative phosphoproteomics of the kinome across the cell cycle. Mol. Cell *31*, 438–448.
9. Novák, B., Tyson, J.J., Gyorffy, B., and Csikasz-Nagy, A. (2007). Irreversible cell-cycle transitions are due to systems-level feedback. Nat. Cell Biol. *9*, 724–728.
10. Hégarat, N., Rata, S., and Hochegger, H. (2016). Bistability of mitotic entry and exit switches during open mitosis in mammalian cells. BioEssays *38*, 627–643.
11. Wu, J.Q., Guo, J.Y., Tang, W., Yang, C.-S., Freel, C.D., Chen, C., Nairn, A.C., and Kornbluth, S. (2009). PP1-mediated dephosphorylation of phosphoproteins at mitotic exit is controlled by inhibitor-1 and PP1 phosphorylation. Nat. Cell Biol. *11*, 644–651.
12. Mochida, S., Ikeo, S., Gannon, J., and Hunt, T. (2009). Regulated activity of PP2A-B55 delta is crucial for controlling entry into and exit from mitosis in Xenopus egg extracts. EMBO J. *28*, 2777–2785.
13. Castilho, P.V., Williams, B.C., Mochida, S., Zhao, Y., and Goldberg, M.L. (2009). The M phase kinase Greatwall (Gwl) promotes inactivation of PP2A/B55delta, a phosphatase directed against CDK phosphosites. Mol. Biol. Cell *20*, 4777–4789.
14. Mochida, S., Maslen, S.L., Skehel, M., and Hunt, T. (2010). Greatwall phosphorylates an inhibitor of protein phosphatase 2A that is essential for mitosis. Science *330*, 1670–1673.
15. Gharbi-Ayachi, A., Labbé, J.-C., Burgess, A., Vigneron, S., Strub, J.-M., Brioudes, E., Van-Dorsselaer, A., Castro, A., and Lorca, T. (2010). The substrate of Greatwall kinase, Arpp19, controls mitosis by inhibiting protein phosphatase 2A. Science *330*, 1673–1677.
16. Blake-Hodek, K.A., Williams, B.C., Zhao, Y., Castilho, P.V., Chen, W., Mao, Y., Yamamoto, T.M., and Goldberg, M.L. (2012). Determinants for activation of the atypical AGC kinase Greatwall during M phase entry. Mol. Cell. Biol. *32*, 1337–1353.
17. Heim, A., Konietzny, A., and Mayer, T.U. (2015). Protein phosphatase 1 is essential for Greatwall inactivation at mitotic exit. EMBO Rep. *16*, 1501–1510.
18. Ma, S., Vigneron, S., Robert, P., Strub, J.-M., Cianferani, S., Castro, A., and Lorca, T. (2016). Greatwall dephosphorylation and inactivation upon mitotic exit is triggered by PP1. J. Cell Sci. *129*, 1329–1339.
19. Rogers, S., Fey, D., McCloy, R.A., Parker, B.L., Mitchell, N.J., Payne, R.J., Daly, R.J., James, D.E., Caldon, C.E., Watkins, D.N., et al. (2016). PP1 initiates the dephosphorylation of MASTL, triggering mitotic exit and bistability in human cells. J. Cell Sci. *129*, 1340–1354.
20. Hégarat, N., Vesely, C., Vinod, P.K., Ocasio, C., Peter, N., Gannon, J., Oliver, A.W., Novák, B., and Hochegger, H. (2014). PP2A/B55 and Fcp1

regulate Greatwall and Ensa dephosphorylation during mitotic exit. PLoS Genet. *10*, e1004004.

21. Zhao, Y., Haccard, O., Wang, R., Yu, J., Kuang, J., Jessus, C., and Goldberg, M.L. (2008). Roles of Greatwall kinase in the regulation of cdc25 phosphatase. Mol. Biol. Cell *19*, 1317–1327.
22. Burgess, A., Vigneron, S., Brioudes, E., Labbé, J.-C., Lorca, T., and Castro, A. (2010). Loss of human Greatwall results in G2 arrest and multiple mitotic defects due to deregulation of the cyclin B-Cdc2/PP2A balance. Proc. Natl. Acad. Sci. USA *107*, 12564–12569.
23. Álvarez-Fernández, M., Sánchez-Martínez, R., Sanz-Castillo, B., Gan, P.P., Sanz-Flores, M., Trakala, M., Ruiz-Torres, M., Lorca, T., Castro, A., and Malumbres, M. (2013). Greatwall is essential to prevent mitotic collapse after nuclear envelope breakdown in mammals. Proc. Natl. Acad. Sci. USA *110*, 17374–17379.
24. Cundell, M.J., Bastos, R.N., Zhang, T., Holder, J., Gruneberg, U., Novák, B., and Barr, F.A. (2013). The BEG (PP2A-B55/ENSA/Greatwall) pathway ensures cytokinesis follows chromosome separation. Mol. Cell *52*, 393–405.
25. Bishop, A.C., Ubersax, J.A., Petsch, D.T., Matheos, D.P., Gray, N.S., Blethrow, J., Shimizu, E., Tsien, J.Z., Schultz, P.G., Rose, M.D., et al. (2000). A chemical switch for inhibitor-sensitive alleles of any protein kinase. Nature *407*, 395–401.
26. Hochegger, H., Dejsuphong, D., Sonoda, E., Saberi, A., Rajendra, E., Kirk, J., Hunt, T., and Takeda, S. (2007). An essential role for Cdk1 in S phase control is revealed via chemical genetics in vertebrate cells. J. Cell Biol. *178*, 257–268.
27. Williams, B.C., Filter, J.J., Blake-Hodek, K.A., Wadzinski, B.E., Fuda, N.J., Shalloway, D., and Goldberg, M.L. (2014). Greatwall-phosphorylated Endosulfine is both an inhibitor and a substrate of PP2A-B55 heterotrimers. eLife *3*, e01695.
28. Potapova, T.A., Sivakumar, S., Flynn, J.N., Li, R., and Gorbsky, G.J. (2011). Mitotic progression becomes irreversible in prometaphase and collapses when Wee1 and Cdc25 are inhibited. Mol. Biol. Cell *22*, 1191–1206.
29. Alvarez-Fernández, M., Sanz-Flores, M., Sanz-Castillo, B., Salazar-Roa, M., Partida, D., Zapatero-Solana, E., Ali, H.R., Manchado, E., Lowe, S., VanArsdale, T., et al. (2018). Therapeutic relevance of the PP2A-B55 inhibitory kinase MASTL/Greatwall in breast cancer. Cell Death Differ. *25*, 828–840.
30. Hutter, L.H., Rata, S., Hochegger, H., and Novák, B. (2017). Interlinked bistable mechanisms generate robust mitotic transitions. Cell Cycle *16*, 1885–1892.
31. Cardelli, L., and Csikász-Nagy, A. (2012). The cell cycle switch computes approximate majority. Sci. Rep. *2*, 656.
32. Müllers, E., Silva Cascales, H., Jaiswal, H., Saurin, A.T., and Lindqvist, A. (2014). Nuclear translocation of cyclin B1 marks the restriction point for terminal cell cycle exit in G2 phase. Cell Cycle *13*, 2733–2743.
33. Feringa, F.M., Krenning, L., Koch, A., van den Berg, J., van den Broek, B., Jalink, K., and Medema, R.H. (2016). Hypersensitivity to DNA damage in antephase as a safeguard for genome stability. Nat. Commun. *7*, 12618.
34. Klebig, C., Korinth, D., and Meraldi, P. (2009). Bub1 regulates chromosome segregation in a kinetochore-independent manner. J. Cell Biol. *185*, 841–858.
35. Ran, F.A., Hsu, P.D., Wright, J., Agarwala, V., Scott, D.A., and Zhang, F. (2013). Genome engineering using the CRISPR-Cas9 system. Nat. Protoc. *8*, 2281–2308.
36. Hsu, P.D., Lander, E.S., and Zhang, F. (2014). Development and applications of CRISPR-Cas9 for genome engineering. Cell *157*, 1262–1278.
37. Kim, S.Y., and Ferrell, J.E., Jr. (2007). Substrate competition as a source of ultrasensitivity in the inactivation of Wee1. Cell *128*, 1133–1145.
38. Trunnell, N.B., Poon, A.C., Kim, S.Y., and Ferrell, J.E., Jr. (2011). Ultrasensitivity in the regulation of Cdc25C by Cdk1. Mol. Cell *41*, 263–274.
39. Toettcher, J.E., Loewer, A., Ostheimer, G.J., Yaffe, M.B., Tidor, B., and Lahav, G. (2009). Distinct mechanisms act in concert to mediate cell cycle arrest. Proc. Natl. Acad. Sci. USA *106*, 785–790.
40. Egea, J.A., Henriques, D., Cokelaer, T., Villaverde, A.F., MacNamara, A., Danciu, D.-P., Banga, J.R., and Saez-Rodriguez, J. (2014). MEIGO: an open-source software suite based on metaheuristics for global optimization in systems biology and bioinformatics. BMC Bioinformatics *15*, 136.
41. Otsu, N. (1979). A threshold selection method from gray-level histograms. IEEE Trans. Syst. Man Cybern. *9*, 62–66.
42. Atherton, T.J., and Kerbyson, D.J. (1993). The coherent circle Hough transform. In Proceedings of the British Machine Vision Conference, J. Illingworth, ed. (British Machine Vision Association), pp. 27.1–27.10.

STAR★METHODS

KEY RESOURCES TABLE

Reagent	Source	Identifier
Antibodies		
Monoclonal anti mouse Cyclin B1, (GNS11)	Thermofisher	Cat# MA5-14327/ lot SG2418437A; RRID: AB_10980440
Monoclonal anti mouse Cyclin A2, (E23.1)	Abcam	Cat# ab38; RRID: AB_304084
Monoclonal anti mouse Cyclin B2, (A-2)	Santa Cruz	Cat# sc-28303; RRID: AB_627340
Polyclonal anti goat Lamin B (c-20)	Santa Cruz	Cat# SC 6216/ lot J2314; RRID: AB_648156
Monoclonal anti rabbit Phospho-CDK substrate motif (k/h pSP)	Cell Signaling	Cat# 94775/ lot 2; RRID: AB_2714143
Polyclonal anti rabbit pY15	Cell Signaling	Cat# 91115/ lot 8; RRID: AB_331460
Polyclonal anti mouse MASTL	Sigma	Cat# HPA054273-10ul/ lot R72184; RRID: AB_2682433
Monoclonal anti mouse CDK1 (A17)	Abcam	Cat# Ab18/ lot GR249349-1; RRID: AB_2074906
Monoclonal anti mouse c-Myc (9E10)	Abcam	Cat# Ab32/ GR310953-3c; RRID: AB_303599
Monoclonal anti mouse Histone H3 pS10 (6.6.2)	Millipore	Cat# 05-499/ lot 2465188; RRID: AB_309763
Polyclonal anti rabbit Tubulin	Abcam	Cat# ab126165/ lot GR106527-3; RRID: AB_11129937
Monoclonal anti rabbit GAPDH (6C5)	Abcam	Cat# ab8245/ lot 1903516; RRID: AB_2107448
Donkey anti rabbit Alexa Fluor 647	Invitrogen	Cat# A31573/ lot 1820538; RRID: AB_2536183
Donkey anti mouse Alexa Fluor 488	Invitrogen	Cat# A21202/ lot 440197; RRID: AB_141607
Donkey anti goat Alexa Fluor 594	Invitrogen	Cat# A11058/ lot 20047670; RRID: AB_2534105
Goat anti rabbit immunoglobulins HRP	Dako	Cat# P0448/ lot 20030309; RRID: AB_2617138
Goat anti mouse immunoglobulins HRP	Dako	Cat# P0447/ lot GR3185172-3; RRID: AB_2617137
Chemicals		
1NMPP1	CalBiochem	Cat# 529581
STLC ((+)-S-Trityl-L-cysteine	Sigma	Cat# 164739
MG132	Sigma	Cat# 474787
Doxycycline	Sigma	Cat# D1822
MK-1775	Selleckchem	Cat# S1525
Dharmafect 1 transfection reagent	Dharmacon	Cat# T-2001-01
Greatwall siRNA	Quiagen	siRNA ID MASTL 06
Edu ClickiT Labeling Kit	Thermo Fisher	Cat# C10337
sirDNA	Spirochrome	Cat# CHF280.00
Experimental Models: Cell Lines		
HeLa cdk1as	Bill Earnshaw	HeLa_as
U2OS cdk1as	Helfrid Hochegger	U2OS_as
MDA MB 231 Greatwall/Cas9	Marcos Malumbres	[23]
Recombinant DNA		
XLcdk1F80GP2APuro in pcDNA 3.1	Bill Earnshaw	Cdl1as_Puro
XLcdk1F80GP2AZeo in pcDNA 3.1	Bill Earnshaw	Cdk1as_Zeo
CyclinB-GFPP2ANeo targeting construct	Helfrid Hochegger	CyclinB-_GFPP2ANeo
cdk1gRNA in pSpCas9(BB)-2A-Puro	Helfrid Hochegger	Cdk1 gRNA
Software and Algorithms		
G2/M model	Bela Novak	see STAR Methods
Phase contrast segmentation Code	Anotida Madzvamuse	https://figshare.com/s/9bf30d093d14fef64ca0

CONTACT FOR REAGENT AND RESOURCE SHARING

Further information and requests for resources and reagents should be directed to and will be fulfilled by the Lead Contact, Helfrid Hochegger (hh65@sussex.ac.uk).

EXPERIMENTAL MODEL AND SUBJECT DETAILS

General cell line culture conditions

U2OS, HeLa and MDA MB 231 cells were cultured in Dulbecco's modified Eagle Medium supplemented with 10% FCS, 2 mM L-glutamine, 100 U/ml penicillin and 0.1 mg/ml streptomycin in a 37°C, 5% CO2 incubator.

HeLa cdk1as cells (Human cervical cancer cell line, female)

HeLa Cdk1as were established by stably expressing Cdk1-F80G-myc using a T2A linked puromycin selection cassette cloned into pcDNA3.1, in HeLa MKK1 cells (A gift from Patrick Meraldi [34],). Endogenous Cdk1 was then removed by CRISPR nuclease targeting using pX459 Cas9T2Apuro [35], target sequence (5′ATTTCCCGAATTGCAGTACTAGG 3′).

HeLa cdk1as cells with GFP tagged endogenous Cyclin B

The Cyclin B1 gRNA was designed in the vicinity of the C-terminal of the coding sequence of Cyclin B1 using Benchling CRISPR tool (https://benchling.com/). The sequences 5′ ACTAGTTCAAGAT-TTAGCCA 3′ were introduced into the vector pSpCas9(BB)-2A-Puro (PX459, acquired from Feng Zhang via Addgene (plasmid # 48139)) V2.0 following the protocol described in [36]. To make the targeting template, Gibson assembly was used to assemble into NotI-digested pAAV-CMV vector (gift from Stephan Geley, University of Innsbruck, Austria) the fragments in the following order: the upstream targeting region (954bp), a linker (5′ CGCCTCAGCGGCAT CAGCTGCAGGAGCTGGAGGTGCATCTGGCTCAGCGG-CAGG 3′), mEmerald, P2A-neomycin and the downstream targeting region (824bp). To get CRISPR-resistant constructs, the sequences ACTAGTTCAAGATTTAGCCAAGG were mutated to AtTAGTc CAgGAccTAGCtAAaG. Mutations (lowercase letters) are silent and preferential codon usage was taken into account. Cas9-gRNA expressing and targeting plasmid were co-transfected in HeLa cdk1as cells using Fugene (Promega) according to manufacturers recommended protocol and integrants were selected in 200 μg/ml G418. And confirmed by Immuno-blotting.

U2OS cdk1as cells (Human bone osteosarcoma epithelial cell, female)

U2OS cells were obtained from the GDSC tisscue culture facility and previously verified by ACTT's cell line authentication service. XLcdk1as expression and knock out of endogenous Cdk1 was established as for HeLa cdk1as cells, except that 5′AATCCATGTACTGACCAGGAGGG 3′ was used as the gRNA target sequence.

MDA MB 231 Cells expressing inducible Greatwall-Cas9-gRNA (Human triple negative breast cancer cell line, female)

These triple negative breast cancer cells express a lentiviral Tet inducible Cas9/gRNA targeting Greatwall [29]. Induction of Cas9 by 1μg/ml Doxycyclin results in Greatwall knock outs and depletion of the protein within 3 days (see Immunoblot confirmation in Figure S4).

METHOD DETAILS

Modeling of mitotic entry and exit

We used nonlinear ordinary differential equations (ODEs) to describe the kinetics of Cdk1:CycB and PP2A:B55 regulation. The ODEs describe the rate of change of the concentration of proteins in the regulatory pathways with respect to time. We used exclusively law of mass action kinetics, approximating enzyme catalyzed reactions by second-order kinetics, ignoring the explicit enzyme–substrate complex (except for pENSA bound to PP2A:B55). Rate constants and rate functions are abbreviated by '*k*' and '*V*', respectively, with subscripts referring to the enzyme and the substrate of the reaction.

Based on the experimental data the model should have some fundamental qualitative features. There should be bistability in the control, Wee1 inhibition, and Gwl depletion cases, with reduced bistability in the latter two. Bistabilty should be diminished in the combined Wee1 inhibition and Gwl depletion case.

Based on the 20 hour G2 block and use of proteasome inhibitor, we can assume that the protein levels are unchanged following the G2 block. This is particularly important for CycB, as well as proteins that are normally degraded during mitotic progression.

A generic substrate is phosphorylated by Cdk1:CycB and dephosphorylated by PP2A:B55:

$$\frac{d[Subp]}{dt} = k_{Cdk1,Sub} \cdot V_{Cdk1} \cdot ([Sub_{Tot}] - [Subp]) - k_{B55,Sub} \cdot [PP2AB55] \cdot [Subp]$$

Cdk1:CycB is dephosphorylated (and activated) by Cdc25 and phosphorylated (and inactivated) by Wee1:

$$\frac{d[CycB:Cdk1]}{dt} = V_{Cdc25} \cdot ([CycB_{Tot}] - [CycB:Cdk1]) - V_{Wee1} \cdot [CycB:Cdk1]$$

PP1 is inactivated when phosphorylated by Cdk1. PP1 reactivates itself *in trans*:

$$\frac{d[PP1]}{dt} = (k_{aPP1} + k_{aPP1a} \cdot [PP1]) \cdot ([PP1_{Tot}] - [PP1]) - (k_{iPP1} + k_{Cdk1,PP1} \cdot V_{Cdk1}) \cdot [PP1]$$

ENSA is phosphorylated by active Gwl and dephosphorylated by B55 when in complex with the phosphatase:

$$\frac{d[pENSA_{Tot}]}{dt} = V_{Gwl} \cdot ([ENSA_{Tot}] - [pENSA_{Tot}]) - k_{catB55} \cdot [pENSA : B55]$$

Gwl is phosphorylated (and activated) by Cdk1:CycB and Cdk2:CycA. Gwl is dephosphorylated (and inactivated) by PP2A:B55, PP1, and a constitutive phosphatase:

$$\frac{d[Gwlp]}{dt} = (k_{Cdk1,Gwl} \cdot V_{Cdk1} + k_{Cdk2,Gwl} \cdot [Cdk2_{Tot}]) \cdot ([Gwl_{Tot}] - [Gwlp]) - (k_{B55,Gwl} \cdot [PP2AB55] + k_{PP1,Gwl} \cdot [PP1] + k_{PPX,Gwl}) \cdot [Gwlp]$$

PP2A:B55 associates with phosphorylated ENSA, titrating the phosphatase away from other substrates. PP2A:B55 dephosphorylates pENSA to which it is bound:

$$\frac{d[PP2AB55]}{dt} = (k_{dis} + k_{catB55}) \cdot [pENSA : B55] - k_{ass} \cdot [PP2AB55] \cdot ([pENSA_{Tot} - [pENSA : B55]])$$

Wee1 is phosphorylated (and inactivated) by Cdk1:CycB and Cdk2:CycA. Wee1 is dephosphorylated (and activated) by PP2A:B55 and a constitutive phosphatase:

$$\frac{d[Wee1]}{dt} = (k_{PPX,Y15} + k_{BB5,Wee1} \cdot [PP2AB55]) \cdot [Wee1p] - (k_{Cdk1,Wee1} \cdot V_{Cdk1} + k_{Cdk2,Wee1} \cdot [Cdk2_{Tot}]) \cdot [Wee1]$$

We include a second inhibitory phosphorylation of Wee1 with the same rate constants for each phosphosite [37]:

$$\frac{d[Wee1pp]}{dt} = (k_{Cdk1,Wee1} \cdot V_{Cdk1} + k_{Cdk2,Wee1} \cdot [Cdk2_{Tot}]) \cdot [Wee1p] - (k_{PPX,Y15} + k_{B55,Wee1} \cdot [PP2AB55]) \cdot [Wee1pp]$$

Cdc25 is phosphorylated (and activated) by Cdk1:CycB and Cdk2:CycA. Cdc25 is dephosphorylated (and inactivated) by PP2A:B55 and a constitutive phosphatase:

$$\frac{d[Cdc25]}{dt} = (k_{PPX,Y15} + k_{B55,Cdc25} \cdot [PP2AB55]) \cdot [Cdc25p] - (k_{Cdk1,Cdc25} \cdot V_{Cdk1} + k_{Cdk2,Cdc25} \cdot [CycA_{Tot}]) \cdot [Cdc25]$$

We include a second activating phosphorylation of Cdc25 [38] with the same rate constants for each phosphosite:

$$\frac{d[Cdc25pp]}{dt} = (k_{Cdk1,Cdc25} \cdot V_{Cdk1} + k_{Cdk2,Cdc25} \cdot [CycA_{Tot}]) \cdot [Cdc25p] - (k_{PPX,Y15} + k_{B55,Cdc25} \cdot [PP2AB55]) \cdot [Cdc25pp]$$

Cdk1 is inhibited by 1NM-PP1 (Inh_{Cdk1}), which in steady state provides the following rate function:

$$V_{Cdk1} = \frac{[CycB : Cdk1]}{1 + \frac{Inh_{Cdk1}}{K_d}}$$

Conservation of total PP2A:B55: $[pENSA : B55] = [B55_{Tot}] - [PP2AB55]$
Conservation of Wee1: $[Wee1p] = [Wee1_{Tot}] - [Wee1] - [Wee1pp]$
Conservation of Cdc25: $[Cdc25p] = [Cdc25_{Tot}] - [Cdc25] - [Cdc25pp]$
Wee1 and Cdc25 activities (rate functions) are the sum of their less- and more-active forms:

$$V_{Wee1} = k_{Wee1S} \cdot ([Wee1_{Tot}] - [Wee1]) + k_{Wee1F} \cdot [Wee1]$$

$$V_{Cdc25} = k_{Cdc25S} \cdot ([Cdc25_{Tot}] - [Cdc25pp]) + k_{Cdc25F} \cdot [Cdc25pp]$$

Gwl rate function depends on the phosphorylated form: $V_{Gwl} = k_{Gwl,ENSA} \cdot [Gwlp]$

This system of differential and algebraic equations was solved with numerical integration, giving the protein concentrations in time. The model output can be interpreted with bifurcation diagrams, which depict the steady state response of a variable with respect to a changing parameter value. This allows for comparison with the end-point experiments in Figure 3B. For Wee1 inhibition, set the parameters k_{Wee1S} and k_{Wee1F} to 0. For Gwl siRNA, set the $[Gwl_{Tot}]$ parameter to 0. For Figure S4A set $Inh_{Cdk\ 1} = 0.5\ \mu M$.

The experimental results are presented as ‘mitotic cells’, whereas the model has ‘substrate phosphorylation’. To directly compare the two, we chose a 30% maximal substrate phosphorylation threshold for the mitotic state, which was based on our immuno-fluorescence quantifications. The bifurcation diagrams in Figure 3C can be compared with the experimental results in this way.

Mathematical modeling of the variation within a population of cells

The nonlinear ordinary differential equations described above can describe the behavior of a single cell. To capture the variation observed between cells in the experiments heterogeneity has to be introduced into the model. We decided to simulate a log-normal distribution of total CycB level for the population of cells (Figure S3F) based on [39] with median 8.18 AU and standard deviation 4.31 AU. Since the total CycB level is assumed to remain constant after release from the G2 block, this involved changing just one parameter for each simulation. Simulating the experimental protocol *in silico* gave the results in Figure 3D, after parameter fitting.

Estimation of the kinetic parameters of the model

Since the intracellular concentrations of proteins are largely unknown (with few exceptions), we have chosen the total concentrations of the following proteins (CycA:Cdk2, PP1, ENSA, Gwl, Wee1, Cdc25 and Sub) as one arbitrary unit, except $B55_{Tot}$ = 0.25 and $CycB_{Tot}$ = 8.18 (see below). Our zero- and second-order rate constants can be immediately converted into nM min^{-1} and nM^{-1} min^{-1} dimensions in view of protein concentrations in nM. We built a customised objective function within the parameter fitting routine MEIGO [40] to parameterise the model. An initial parameterisation was performed using the 50% levels for each of the four experimental conditions. This determined a median cyclin B level. Next, the log-normal cyclin B distribution was used to fit all of the experimental data, and this time the variance of the cyclin B distribution was fitted. We used the values in [27] as initial values for the binding and activity of PP2A:B55 toward pENSA. After fitting, the catalytic and association (after scaling) values were within 3–5-fold of the literature values. All the parameters and initial conditions are in the tables below.

Parameter values used in the model.

Name	Description	Value
Inh_{Cdk1}	Level of 1NM-PP1 added	0–2 μM
k_{aPP1}	Constitutive dephosphorylation and thereby activation of PP1	0.01 min^{-1}
k_{aPP1a}	Dephosphorylation of PP1 by dephosphorylated PP1 *in trans*	0.70 min^{-1}
k_{iPP1}	Constitutive phosphorylation and hence inactivation of PP1	0.002 min^{-1}
$k_{Cdk1,PP1}$	Phosphorylation of PP1 by Cdk1:CycB	0.75 min^{-1}
$k_{PP1,Gwl}$	Dephosphorylation of Gwl by PP1	18.47 min^{-1}
k_{ass}	Association of phosphorylated ENSA with PP2A:B55	617 min^{-1}
k_{dis}	Dissociation of the pENSA:PP2A:B55 complex	0.009 min^{-1}
k_{catB55}	Dephosphorylation of pENSA when in complex with PP2A:B55	1 min^{-1}
$k_{Gwl,ENSA}$	Phosphorylation of ENSA by Gwl	21 min^{-1}
$k_{PPX,Gwl}$	Basal dephosphorylation of Gwl	0.16 min^{-1}
$k_{Cdk1,Sub}$	Phosphorylation of the substrate by Cdk1:CycB	0.008 min^{-1}
$k_{Cdk1,Gwl}$	Phosphorylation of Gwl by Cdk1:CycB	0.24 min^{-1}
$k_{B55,Gwl}$	Dephosphorylation of Gwl by PP2A:B55	496 min^{-1}
$k_{B55,Sub}$	Dephosphorylation of the substrate by PP2A:B55	0.06 min^{-1}
$k_{Cdk2,Gwl}$	Phosphorylation of Gwl by Cdk2	0.19 min^{-1}
k_{Cdc25S}	Dephosphorylation of Y15 of Cdk1 by unphosphorylated Cdc25	0.005 min^{-1}
k_{Cdc25F}	Dephosphorylation of Y15 of Cdk1 by phosphorylated Cdc25	0.94 min^{-1}
k_{Wee1S}	Phosphorylation of Y15 of Cdk1 by phosphorylated Wee1	0.005 min^{-1}
k_{Wee1F}	Phosphorylation of Y15 of Cdk1 by unphosphorylated Wee1	47 min^{-1}
$k_{Cdk1,Wee1}$	Phosphorylation of Wee1 by Cdk1:CycB	1.31 min^{-1}
$k_{Cdk1,Cdc25}$	Phosphorylation of Cdc25 by Cdk1:CycB	1.31 min^{-1}
$k_{PPX,Y15}$	Dephosphorylation of Wee1 and Cdc25 by a constitutive phosphatase	0.005 min^{-1}
$k_{Cdk2,Wee1}$	Phosphorylation of Wee1 by Cdk2	0.11 min^{-1}
$k_{Cdk2,Cdc25}$	Phosphorylation of Cdc25 by Cdk2	0.11 min^{-1}
$k_{B55,Wee1}$	Dephosphorylation of Wee1 by PP2A:B55	0.55 min^{-1}
$k_{B55,Cdc25}$	Dephosphorylation of Cdc25 by PP2A:B55	0.55 min^{-1}
K_d	Dissociation constant for 1NM-PP1 with Cdk1	0.025 min^{-1}

Initial conditions correspond to interphase arrest.

Dynamic variable	Initial value
Subp	0
CycB : Cdk1	0
PP1	1
$pENSA_{Tot}$	0
Gwlp	0

(*Continued on next page*)

Continued	
Dynamic variable	Initial value
PP2AB55	0.25
Wee1	1
Wee1pp	0
Cdc25	1
Cdc25pp	0

XPPAUT code for the bifurcation diagrams of Figure 3C, Figure S3E, and Figure S4A.

```
Subp' = kcBc1Sub*CycBCdk1/((1 + (InhCDK/Kd)))*(SubT-Subp) - kB55Sub*PP2AB55*Subp
CycBCdk1' = V25*(CycBCdk1T - CycBCdk1) - Vwee*CycBCdk1
PP1' = (kapp1 + kapp1a*PP1)*(PP1T - PP1) - (kipp1 + kipp1C*CycBCdk1/((1 + (InhCDK/Kd))))*PP1
pENSAt' = VGwl*(ENSAtot - pENSAt) - kcatB55*Complex
Gwlp' = (kcBc1G*CycBCdk1/((1 + (InhCDK/Kd))) + kcAc2G*CycACdk2T)*(Gwtot - Gwlp) - (kB55G*PP2AB55 + kppxGwl +
kPP1Gw*PP1)*Gwlp
PP2AB55' = kdis*Complex + kcatB55*Complex - kass*PP2AB55*(pENSAt - Complex)
Complex = B55tot - PP2AB55
Wee1' = (kppxY15 + kB55W1*PP2AB55)*Wee1p - (kcBc1W1*CycBCdk1/((1 + (InhCDK/Kd))) \
+ kcAc2W1*CycACdk2T)*Wee1
Wee1pp' = (kcBc1W1*CycBCdk1/((1 + (InhCDK/Kd))) + kcAc2W1*CycACdk2T)*Wee1p - \
(kppxY15 + kB55W1*PP2AB55)*Wee1pp
Wee1p = 1 - Wee1 - Wee1pp
Cdc25' = (kppxY15 + kB5525*PP2AB55)*Cdc25p - (kcBc125*CycBCdk1/((1 + (InhCDK/Kd))) + kcAc225*CycACdk2T)*Cdc25
Cdc25pp' = (kcBc125*CycBCdk1/((1 + (InhCDK/Kd))) + kcAc225*CycACdk2T)*(Cdc25p) - \
(kppxY15 + kB5525*PP2AB55)*Cdc25pp
Cdc25p = 1 - Cdc25 - Cdc25pp
Vwee = (kweeS*(1-Wee1) + kweeF*Wee1)
V25 = k25S*(1-Cdc25pp) + k25F*Cdc25pp
VGwl = kGwENSA*Gwlp
init Subp = 0, CycBCdk1 = 0, PP1 = 1, pENSAt = 0, Gwlp = 0, PP2AB55 = 0.25, Wee1 = 1, Wee1pp = 0, Cdc25 = 1, Cdc25pp = 0
p InhCDK = 1, CycBCdk1T = 8.1808, CycACdk2T = 1.0000, PP1T = 1.0000, kapp1 = 0.0115
p kapp1a = 0.7054, kipp1 = 0.0018, kipp1C = 0.7549, kPP1Gw = 18.4724
p ENSAtot = 1.0000, B55tot = 0.2500, SubT = 1.0000, kass = 617.2807
p kdis = 0.0088, kcatB55 = 1.0338, kGwENSA = 20.8811, kppxGwl = 0.1560, kcBc1Sub = 0.0080
p kcBc1G = 0.2393, Gwtot = 1.0000, kB55G = 496.5636, kB55Sub = 0.0593
p kcAc2G = 0.1916, k25S = 0.0050, k25F = 0.9411, kweeS = 0.0050, kweeF = 47.2937
p kcBc1W1 = 1.3132, kcBc125 = 1.3132, kppxY15 = 0.0050, kcAc2W1 = 0.1096
p kcAc225 = 0.1096, kB55W1 = 0.5511, kB5525 = 0.5511, Kd = 0.025
@ total = 1000,dt = 0.1,meth = STIFF,xlo = 0,xhi = 100,ylo = 0,yhi = 1
@ NTST = 15,NMAX = 1000000,NPR = 10000,DS = −0.001
@ DSMAX = 0.005,DSMIN = 0.001,PARMIN = 0,PARMAX = 10
@ AUTOXMIN = 0,AUTOXMAX = 0.8,AUTOYMIN = 0,AUTOYMAX = 1
done
```

Preparation of total cell extracts, immunoblotting

For cell lysate preparation 5 × 10E5 cells were washed once in PBS and lysed in 50 μL ECB buffer (50 mM Tris pH 7.5, 120 mM NaCl, 0.5% NP40, 1 mM EDTA, 0.05% β-mercaptoethanol and protease (1 tablet per 50 ml) and phosphatase (1 tablet per 10 ml) inhibitors, and incubated on ice for 20 min. Cells were sonicated. Protein concentrations were determined by Bradford method and lysates were equalized for protein concentration using ECB buffer. Samples were then resuspended in 1 × SDS–PAGE sample buffer (12.5 mM Tris–HCl pH 6.8, 1.4% (w/v) SDS, 4% sucrose (w/v), 0.002% (w/v) bromophenyl blue, 0.4 mM β-mercaptoethanol). Samples were then analyzed by western blotting.

siRNA transfections

Cells were reverse transfected with 20 nM siRNA at a density of 0.4 × 105 cells/ml using Dharmafect 1 transfection reagent (Dharmacon) following the manufacturers' protocol. Eight hours following transfection the medium was changed and cells were

synchronized with 1NM-PP1 24 hours later. Control and Greatwall siRNA (MASTL 06, 5′ACGCCTTATTCTAGCAAATTA3′) were purchased from QIAGEN.

FACS analysis

Cells were incubated with 10 μM of EdU for 1h before being harvested and fixed in 70% ethanol. Next, EdU was labeled with the fluorophore Alexa 647 using Click-iT EdU Imaging Kit (Invitrogen) and DNA were stained with 5 μg/mL of propidium iodide (Fluka) and 150 μg/mL of RNaseA (Sigma-Aldrich). Facs profiles were obtained from data acquired on Accuri C6 Flow cytometer (BD Biosci-ences).

Immunofluorescence and live cell imaging

HeLa cdk1as cells were grown on coverslips and fixed for 10 min in 3.7% formalde-hyde, rinsed 3 times in PBS. Coverslips were then rinsed in PBS and cells permeabilized in PBS-0.1% NP40. Cells were blocked in 2% BSA for 10 min and probed with primary antibodies (as indicated in figure legends) for 40 min. Slides/coverslips were rinsed 4 × in PBS and probed with Alexa secondary antibodies listed for 20 min. Slides/coverslips were then rinsed 4 × in PBS and coverslips were mounted using ProLong® Gold mounting solution containing DAPI (Invitrogen). For image acquisition, we used a Olymnpus IX73 microscope. Imaging was performed using a UPLanS Apo, N.A. 1.43, × 60 oil immersion objective (Olympus), standard filter sets (excitation 360/40; 490/20; 555/28; emission 457/50; 528/38; 617/40) and a Prime sCMOS camera (Photometrics). Z-series of 0.7 μm stacks were acquired using Micromanager software (Version 6.0.8) and images exported as tiff files. Images were analyzed in ImageJ. Regions were manually marked on the image for analysis and analyzed using the ImageJ ROI manager tool. Live cell imaging was performed on the Olympys IX73 microscope equipped with an environmental chamber (Digital Pixel), using either a 0.6NA 40x PlanFL long distance objective suitable for differential interference contracts imaging, or a 20x 0.45NA PlanFL long distance objective designed for phase contrast image acquisition in the wide field channel. Images were acquired using Micromanager software (Version 6.0.8) and recorded by a Prime sCMOS camera at 37C Temperature and 5% CO2. Environmental conditions were maintained by an environmental chamber from Digital Pixel.

Bistability Assay

HeLa cdk1as cells were plated at a density of 7500 cells into 96 well half area high content imaging glass bottom microplates (Corning, 4580). U2OS cdk1as cells were plated in 96 well plates (Perkin Elmer Cell carrier- 96 Ultra) that were coated with 40 μL of a 1/10 dilution in PBS of Collagen1 (Pan Biotech) and left to dry in a tissue culture hood. Before plating cells, collagen was activated by washing with PBS two times. Cells were plated at the same density as HeLa cdk1as cells. 24 hours later medium was changed to medium containing 2 μM 1NM-PP1. Following 20 hour 1NM-PP1 incubation the mitotic exit samples were washed 5 times with medium, following the last wash 50 μl medium containing 25 μM MG231 was added. 90 minutes later the mitotic exit samples were washed in the same way and released in 25 μM MG231 medium. Finally, 50 μl medium with 25 μM MG231 and 2x concentrations of the indicated inhibitors was added. Cells were fixed after a further four hour incubation by adding 10 μl 3.4% Formaldehyde for 10 minutes. Subsequently the fixation medium was removed and resuspended in PBS containing 2 μM Hoechst 33342 (Thermofisher). Imaging was performed on a Olympus IX81 SCANR microscope using a 0.4 NA PlanFL 20x long distance objective, with a camera ORCA-R2(Hamamatsu CCD). Nuclei were segmented using a bespoke SCANR algorithm based on pixel intensity and 100 random nuclei per well were displayed using the SCANR gallery tools and counted manually.

Automated measurement of mitotic index in live cell experiments

We developed a bespoke MATLAB script to count the number of mitotic cells in a time lapse experiment. This is based on estimating the total number of cells in the field of view based on segmentation of the sirDNA stained nuclei using a standard Otsu algorithm [41]. Mitotic cells were detected by the change in intensity in the phase contract channel during cell rounding. To identify the resulting bright halo circles in the segmentation, we employed an algorithm based on a circular Hough Transform [42] and filtered the identified objects by circularity and size.

QUANTIFICATION AND STATISTICAL ANALYSIS

All experiments included at least three independent biological repeats. Sample size per repeat varied between experiments and are indicated in the Figure Legends as well as Mean, Standard Deviation or Median values. Sample size was based on standard practise in cell biological assays and not specifically pre-estimated. P values were calculated using a 1 tail unequal variance t test. For all experiments, samples were not randomized and the investigators were not blinded to the group allocation during experiments and outcome assessment. No exclusion criteria were used and all collected data were used for statistical analysis.

Current Biology, Volume *28*

Supplemental Information

Two Interlinked Bistable Switches Govern Mitotic Control in Mammalian Cells

Scott Rata, Maria F. Suarez Peredo Rodriguez, Stephy Joseph, Nisha Peter, Fabio Echegaray Iturra, Fengwei Yang, Anotida Madzvamuse, Jan G. Ruppert, Kumiko Samejima, Melpomeni Platani, Monica Alvarez-Fernandez, Marcos Malumbres, William C. Earnshaw, Bela Novak, and Helfrid Hochegger

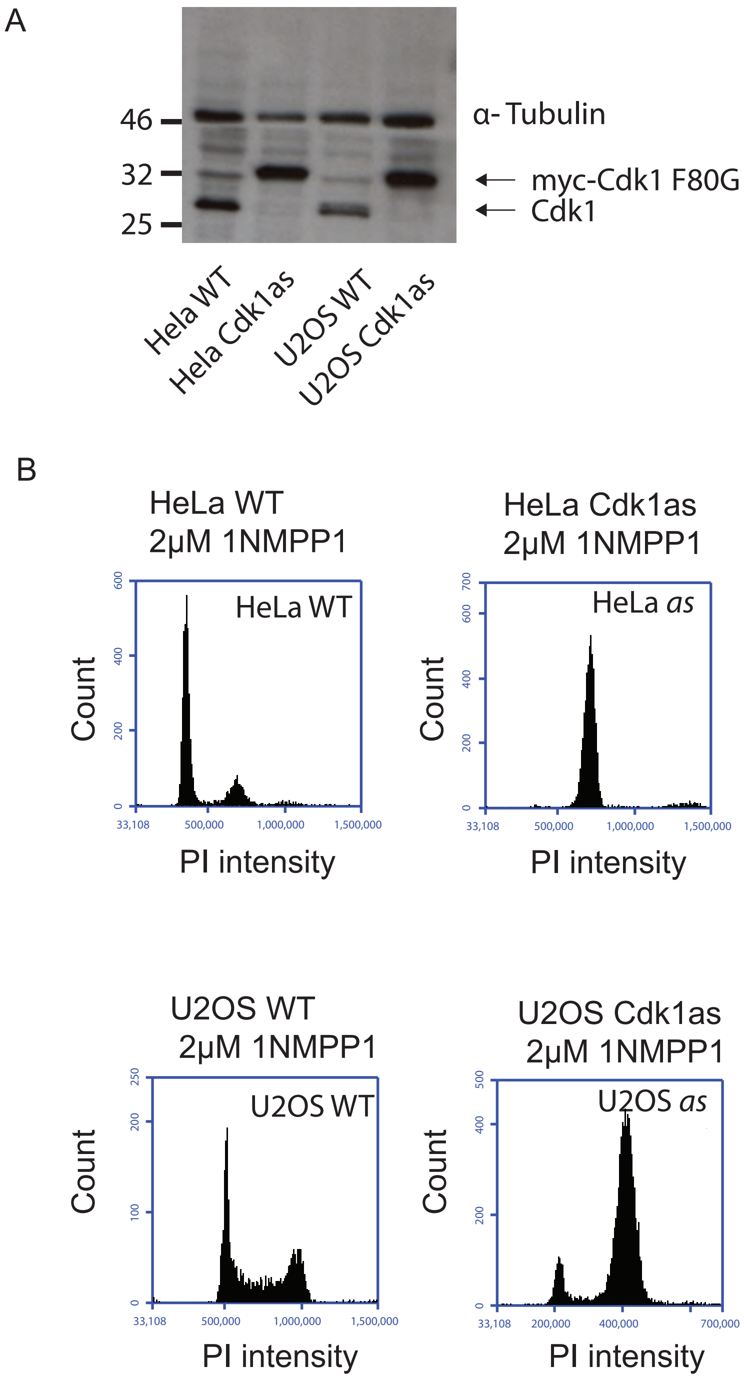

Figure S1 Characterisation of cdk1as cells (Related to Figure 2)
(A) Immuno-blots confirming generation of cdk1as HeLa and U2OS cells. (B) PI staining and FACS analysis of HeLA and U2OS WT and cdk1as cells after 20 hour treatment with 2 µM 1NMPP1.

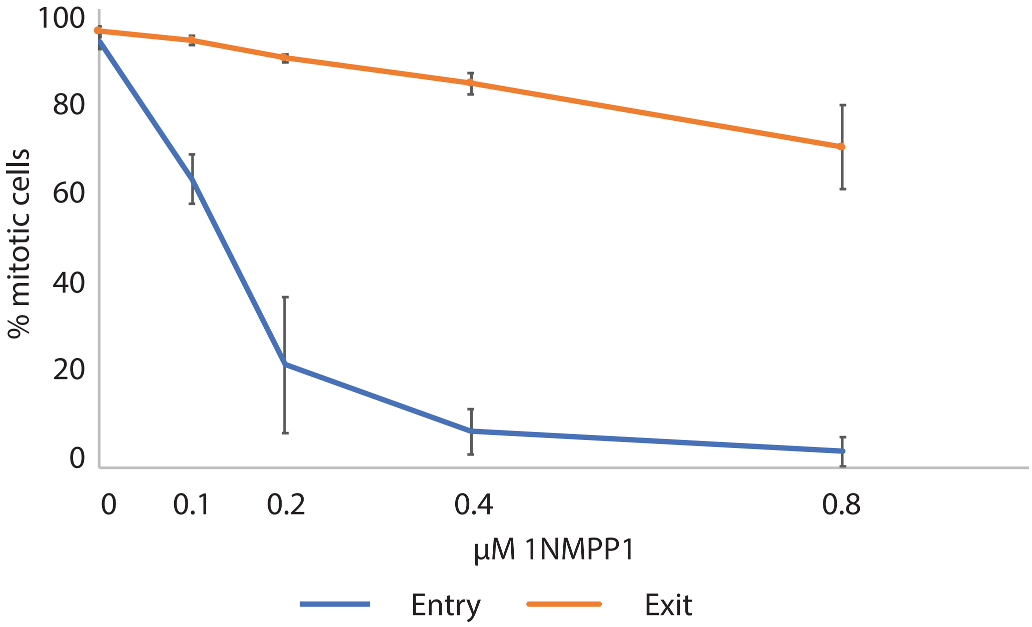

Figure S2 Bistability assay in U2OS cdk1as cells (Related to Figure 2)
Experiments performed as described in Figure 2D in U2OS cdk1as cells (means and stdv of 3 biological repeats are shown, with N=100 per repeat)

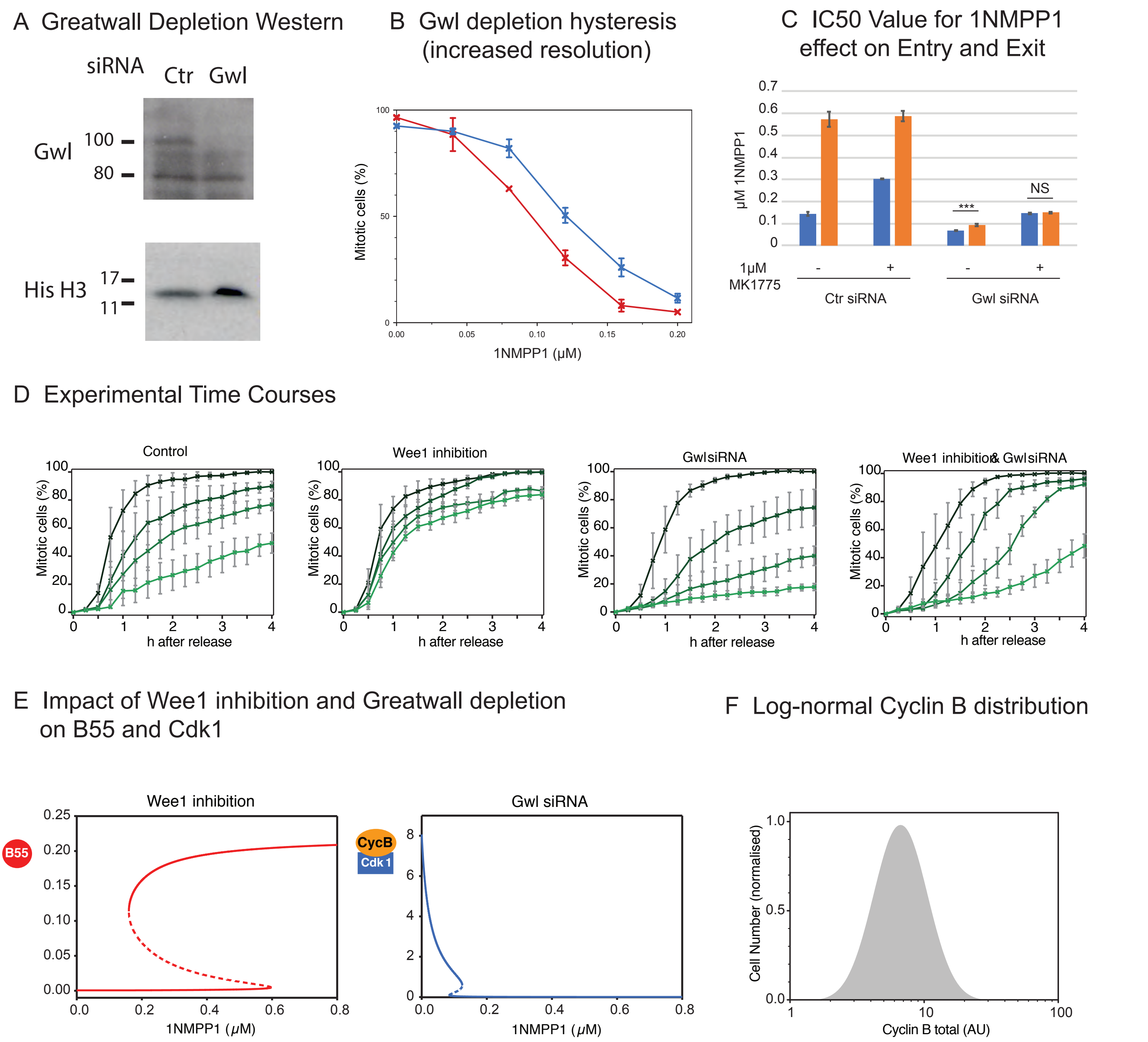

Figure S3 Data supporting bistability assays (related to Figure 3)
(A) Immuno-blots confirming Gwl siRNA knockdown efficiency. HeLa cdk1as cells were probed 48 hours after siRNA transfection. (B) Mitotic entry and exit curves of cdk1as HeLa cells depleted of Gwl at increased resolution of 1NMPP1 concentration. Mean of two repeats plotted, with error bars indicating standard deviation.
(C) Midpoint values of 1NMPP1 response based on experiments in Figure 3B and linear interpolation. Means of 3–4 biological repeats are plotted, with error bars indicating standard deviation.
Statistical significance was assessed by the one-tailed student's t-test.
(D) Dynamics of mitotic entry at increasing 1NMPP1 concentrations (0, 0.1, 0.2, and 0.4 µM) from live-cell imaging analysis in cells subjected to indicated treatment. Graphs represent the mean of three independent experiments and error bars indicate standard error. (E) Simulated signal-response curves for PP2A:B55 activity (left) and Cdk1 activity (right) as a function of Cdk1 inhibitor for the cases of Wee1 inhibition and Gwl depletion, respectively. The bistability of these enzymes gives rise to the hysteresis of Cdk1/PP2A:B55 substrate phosphorylation in Figure 3C (F) Estimated Cyclin B distribution that was used to model the population analysis data shown in Figure 3D, with median 8.18 AU and standard deviation 4.31 AU.

Figure S4

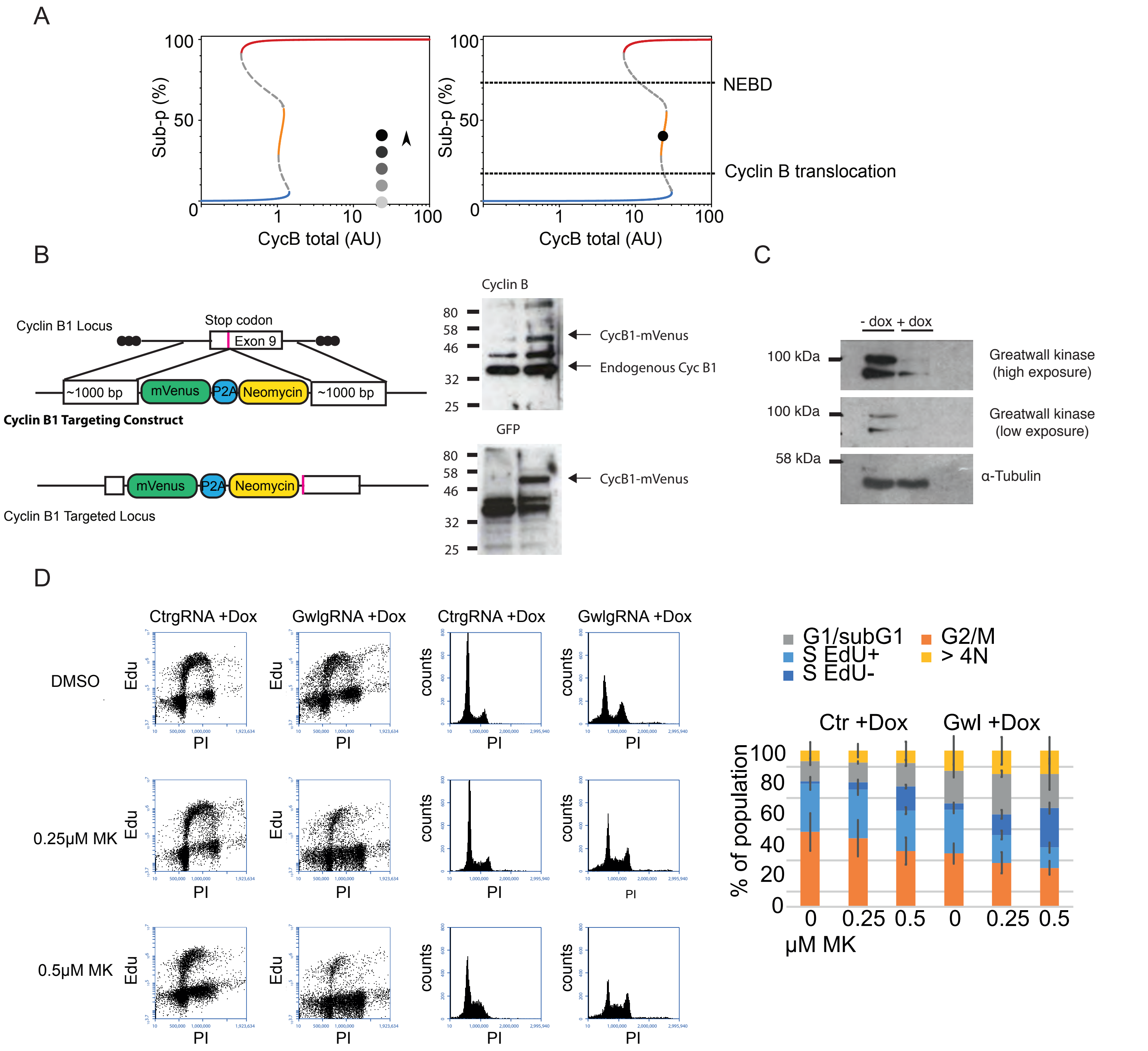

Figure S4 Data supporting prophase experiments and Wee1/Greatwall synergy (Related to Figure 4)
(A) Signal-response curve for cells (filled circles) released from G2 block (left) and after Cdk1 inhibitor (0.5 μM) addition (right). Addition of the right amount of inhibitor at the right time captures some cells at the intermediate steady state. (B) Strategy and confirmation of generation of CyclinB-GFP tagged HeLa cdk1as cells by immune-blotting
(C) Depletion of Greatwall kinase in MDA MB 231 cells three days after induction of GwlgRNA/Cas9. (D) Representative examples and quantification of FACS profiles after PI/Edu staining of Ctr gRNA/Cas9 or Greatwall gRNA/Cas9 expressing MDA MB 231 cells (4 days after addition of Doxycyclin). Cells were treated with indicated concentration of MK1775 for the last 24 hours before Edu labelling. Shown are the means of three biological repeats (N=10000 per repeat), Error bars represent the STDV of three independent experiments.
